# Steering carbon dioxide reduction toward C–C coupling using copper electrodes modified with porous molecular films

**DOI:** 10.1038/s41467-023-36530-z

**Published:** 2023-02-15

**Authors:** Siqi Zhao, Oliver Christensen, Zhaozong Sun, Hongqing Liang, Alexander Bagger, Kristian Torbensen, Pegah Nazari, Jeppe Vang Lauritsen, Steen Uttrup Pedersen, Jan Rossmeisl, Kim Daasbjerg

**Affiliations:** 1 Interdisciplinary Nanoscience Center (iNANO), Gustav Wieds Vej 14, 8000 Aarhus C, Denmark; 2 Novo Nordisk Foundation (NNF) CO2 Research Center, Aarhus University, Gustav Wieds Vej 10C, 8000 Aarhus C, Denmark; 3 Department of Chemistry, University of Copenhagen, Universitetsparken 5, 2100 Copenhagen O, Denmark; 4 Leibniz-Institut für Katalyse, Albert-Einstein-Straße 29a, 18059 Rostock, Germany; 5 Department of Chemistry, Aarhus University, Langelandsgade 140, 8000 Aarhus C, Denmark

**Keywords:** Electrocatalysis, Pollution remediation, Materials for energy and catalysis, Surface assembly

## Abstract

Copper offers unique capability as catalyst for multicarbon compounds production in the electrochemical carbon dioxide reduction reaction. In lieu of conventional catalysis alloying with other elements, copper can be modified with organic molecules to regulate product distribution. Here, we systematically study to which extent the carbon dioxide reduction is affected by film thickness and porosity. On a polycrystalline copper electrode, immobilization of porous bipyridine-based films of varying thicknesses is shown to result in almost an order of magnitude enhancement of the intrinsic current density pertaining to ethylene formation while multicarbon products selectivity increases from 9.7 to 61.9%. In contrast, the total current density remains mostly unaffected by the modification once it is normalized with respect to the electrochemical active surface area. Supported by a microkinetic model, we propose that porous and thick films increase both local carbon monoxide partial pressure and the carbon monoxide surface coverage by retaining in situ generated carbon monoxide. This reroutes the reaction pathway toward multicarbon products by enhancing carbon–carbon coupling. Our study highlights the significance of customizing the molecular film structure to improve the selectivity of copper catalysts for carbon dioxide reduction reaction.

## Abstract (web summary)

This study explores how the activity and selectivity of Cu electrodes for CO_2_ reduction can be tuned with organic films of different porosity and thickness. As a result, the films increase the local CO partial pressures and surface coverages.

## Introduction

The electrochemical carbon dioxide reduction reaction (CO_2_RR) constitutes a carbon-neutral method for manufacturing carbon chemical feedstock and storing electricity from renewable sources such as wind and solar^1,2^. Cu is by far the best electrocatalyst for forming hydrocarbons and oxygenated products^3,4^, although recently low selectivity of longer carbon chains was also observed on nickel-based catalysts^5^. The modest adsorption energy of carbon monoxide (CO), a critical CO_2_RR intermediate, makes it feasible for this intermediate to be further reduced to hydrocarbons on Cu electrodes. This gives rise to numerous distinct products, including both single carbon (C_1_) products [e.g., formate (HCOO^−^), CO, and methane (CH_4_)], and multicarbon (C_2+_) products [e.g., acetate (CH_3_COO^−^), ethylene (C_2_H_4_), ethanol (C_2_H_5_OH), and isopropanol (C_3_H_7_OH)]^6,7^. The C_2+_ products are particularly interesting because of their larger energy density and wider applicability^8^. To switch the reaction pathway from C_1_ to C_2+_, as well as suppress the competitive hydrogen (H_2_) evolution reaction (HER), considerable research has been conducted on adjusting the Cu morphology (e.g., size^9^, grain boundaries^10,11^, and facets^12–15^), oxidation states^16–18^, controlling the dopants (e.g., alloy)^19–22^, and electrolyte (e.g., cations^23,24^ and anions^25^).

In addition, the concept of steering activity and selectivity toward C_2+_ products during CO_2_RR by introducing molecular catalysts or organic compounds containing specific functional groups to tailor the Cu surface is well established^26–30^. These studies ascribe the improved CO_2_RR performance to various phenomena, including electric field strength^31^, stabilization of CO_2_ reduction intermediate, hydrophobicity^32,33^, or higher CO_2_ concentration in the vicinity of the electrode^31,34^. For instance, Sargent and co-workers demonstrated the correlation between the selectivity of C_2_H_4_ and the ratio of atop-bound CO to bridge-bound CO on Cu surfaces by modifying Cu with a library of *N*-arylpyridinium salts with different substituents^35^. Züttel and co-workers revealed that the distinct hydrophobic properties of polymers could change the transport of H_2_O and CO_2_, thus greatly affecting the CO_2_RR activity^36^. Nonetheless, recently, Grubbs and Goddard III found that a tricomponent copolymer-modified Cu electrode rivaled the best performance of existing modified polycrystalline Cu foil catalysts due to not only the electric field induced by the polymer but also the increased porosity of the polymer film for CO_2_ diffusion^31^. This underlines the significance of the physical structure of organic films and animates to further research into understanding the impact of film thickness and porosity of organic films. Such knowledge is pertinent as it would better enable customization of the chemical microenvironment surrounding a Cu catalyst for optimal C_2+_ production.

In this work, we present a systematic study of the impact of film structure on the electrochemical CO_2_RR at Cu surfaces. Inspired by the work of Sargent and co-workers^35^, electrochemical deposition was used to systematically manufacture electrodes containing organic films of varying thicknesses and porosities. Remarkably, a carefully prepared porous and thick 1,1′-di-p-tolyl-1,1′,4,4′-bipyridine (T-bipyridine) film deposited on a polycrystalline Cu electrode can give rise to almost an order of magnitude enhancement of the intrinsic current density of C_2_H_4_. Mechanistic studies highlight the crucial role of the physical structure of the molecular modifier for selectively converting CO_2_ to C_2+_ products. Supported by a microkinetic model, we propose the capability of porous and thick organic films to boost the C–C coupling and C_2+_ product generation. This occurs by establishing a ^*^CO-rich microenvironments close to the Cu surface by retaining and extending the residence time of in situ generated CO.

## Results and discussion

### Electrodes preparation and surface characterization

**Fig. 1:**
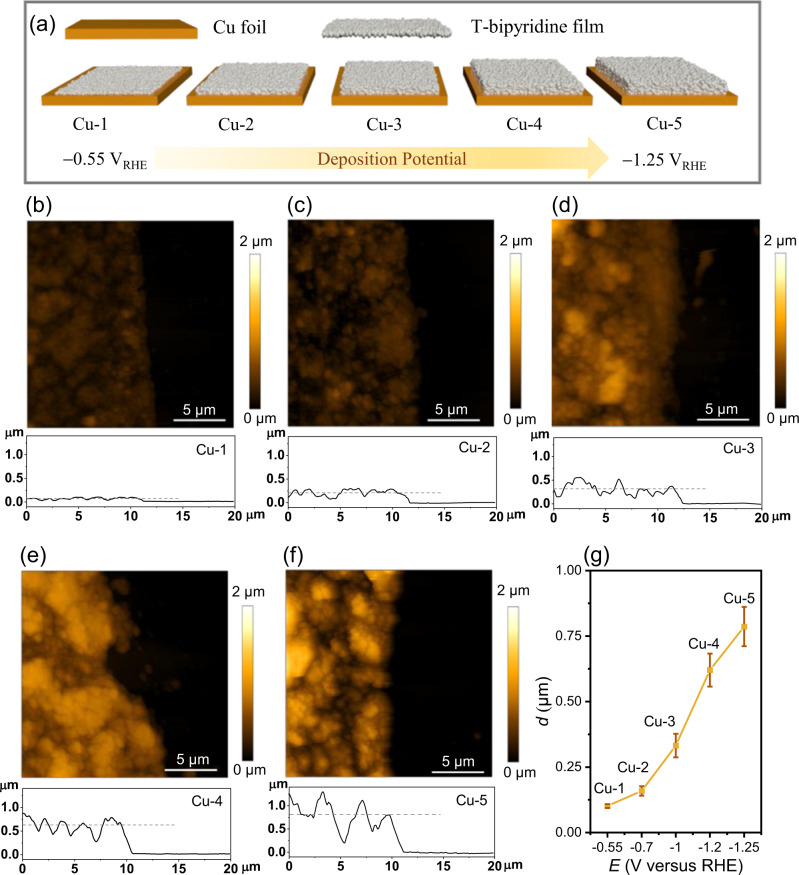
Thickness characterization of of T-bipyridine modified Cu electrodes. **a** Schematic of the five T-bipyridine modified electrodes denoted Cu-n (n = 1−5). **b**−**f** AFM images and cross-sectional profiles of the edge plane (20 × 20 μm^2^) on Cu-n prior to electrolysis. **g** Average thicknesses of films on Cu-n. Error bars correspond to the standard deviation of >15 regions on each sample. Source data are provided as Source Data file.

Following a modified electrodimerization procedure published by Sargent and co-workers^35^, T-bipyridine films were electrodeposited on polished Cu foil electrode by applying five specific potentials in the range of −1.25 to −0.55 V versus reversible hydrogen electrode (RHE) in CO_2_-saturated 0.1 M KHCO_3_. In general, employment of a more negative potential would be anticipated to lead to a relatively larger deposit of T-bipyridine on the Cu surface, and thus a thicker film, as a greater electrochemical driving force is applied. The modified electrodes (Fig. 1a), denoted Cu-n (n = 1–5), were prepared according to the conditions summarized in Table S1.

Atomic force microscopy (AFM) measurements were performed to determine the film thickness, *d*, on the Cu-n plates, made possible by the fact that the electrodes had been partially covered with a piece of polyimide tape during electrochemical deposition of T-bipyridine (Fig. 1b−f). After removal of the tape, this created a distinct step between the exposed Cu substrate (dark, right side) and deposited film (bright, left side); *d* was determined as the mean value based on cross-sectional profiles (white rectangular box) pertaining to >15 different regions on each sample to average thickness fluctuations. Fig. 1g displays a plot of *d* against the applied deposition potential. A distinct correlation exists between these two parameters with *d* increasing from 0.10 ± 0.01 to 0.79 ± 0.08 µm as the applied potential decreases from **−**0.55 to **−**1.25 V versus RHE.

**Fig. 2:**
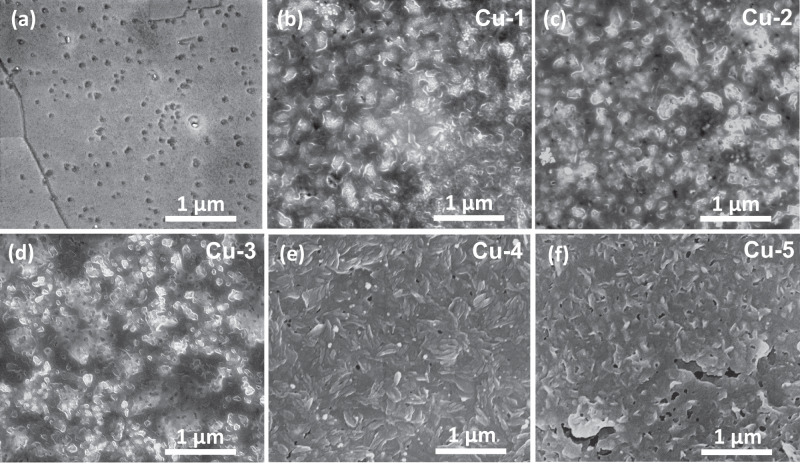
SEM images. **a** pristine Cu and **b**−**f** T-bipyridine modified Cu-n (n = 1−5) electrodes prior to electrolysis.

Next, scanning electron microscopy (SEM) images were captured for all electrodes to assess the film morphology (Fig. 2). Figure 2a shows that the surface of pristine Cu is flat after the initial mechanical and electrochemical polishing. Some inner degrading voids of Cu are exposed on the surface due to non-selective electrochemical corrosion by phosphoric acid, which is consistent with prior observations^13,37^. For the modified electrodes, the T-bipyridine film is evident, and it is becoming more porous and rougher as the deposition potential decreases to more negative values (Fig. 2b−f and S3).

Film porosity (*P*) was evaluated by measuring the volume of pores in each film using AFM for all the as-prepared films once they were contracted by means of drop casting acetone directly onto the organic films. In this manner, the organic film was dissolved before it was rebuilt upon acetone evaporation. Based on the assumptions that (i) the reconstructed film is a dense film without pores and (ii) the reconstructed film is distributed evenly on the electrode surface, *P* can be calculated according to Eq. (1).

1 $$P=\frac{{V}_{{{{{{\rm{pore}}}}}}}}{{V}_{{{{{{\rm{total}}}}}}}}100 \%=\frac{(d-{d}_{{{{{{\rm{recon}}}}}}})A}{d\times A}100\%=\,\frac{(d-{d}_{{{{{{\rm{recon}}}}}}})}{d}100\%$$

Here, *P* is defined as the ratio of pore volume (*V*_pore_) and total volume (*V*_total_), with the latter volume determined from the thickness of the film (*d*) times the area of the electrode (*A*), and the former determined from the thickness difference of the film before and after reconstruction (*d*_recon_) times *A*. Table S2 collects the results, showing that *P* increases as the film thickness increases from Cu-1 (*P* = 55%) to Cu-5 (*P* = 83%). This substantiates our observations from SEM that the film becomes more porous as the applied potential used for the film deposition decreases.

### Electrochemical CO_2_RR

**Fig. 3:**
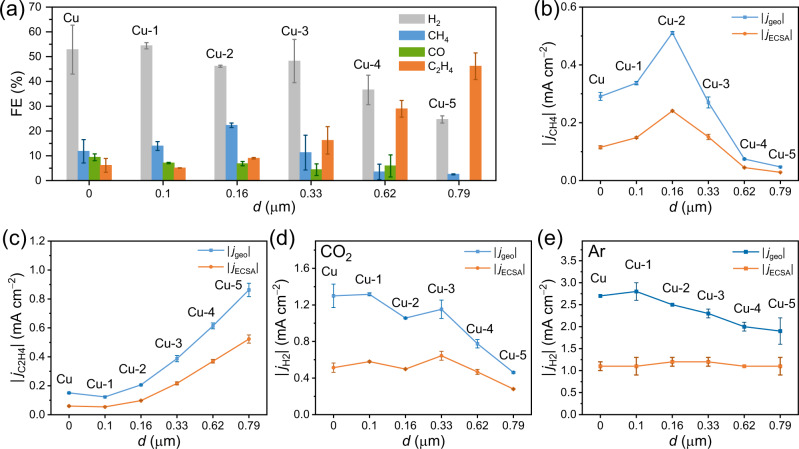
Performance of pristine Cu and Cu-n (n = 1−5) electrodes in CO_2_RR. **a** FEs of H_2_ (gray), CH_4_ (blue), CO (green) and C_2_H_4_ (orange) as function of *d* along with partial |*j*| of **b** CH_4_, **c** C_2_H_4_, **d** H_2_ and **e** H_2_ (under Ar atmosphere with no CO_2_RR) normalized with respect to the geometric surface area (=1.22 cm^2^, blue) and ECSA (orange), respectively, as function of *d*. All bulk electrolysis experiments were conducted at an applied potential of −0.96 V versus RHE for 60 min in CO_2_-saturated or Ar-saturated 0.1 M KHCO_3_, unless noted otherwise. Error bars correspond to the standard deviation of at least two independent measurements. Source data are provided as a Source Data file.

The performance of all fabricated Cu electrodes in CO_2_RR was tested in bulk electrolysis experiments (at −0.96 V versus RHE in CO_2_-saturated 0.1 M KHCO_3_ for 60 min) to evaluate the extent by which the exact film structure would impact the electrocatalytic properties, both with respect to selectivity and activity (Fig. 3a and Table S3). Regarding selectivity, Cu-5 (*d* = 0.79 ± 0.08 µm) demonstrates the highest FE_C2H4_ of 46.1%, with minor CH_4_ production (FE_CH4_ = 2.6%) and surprisingly no CO. The corresponding value of FE_H2_ is 24.7%, showing a significantly lowering of H_2_ production. For comparison, pristine Cu primarily produces H_2_ (FE_H2_ = 52.8%) and C_1_ products (FE_CH4_ = 11.8%, FE_CO_ = 9.4%), along with minor amounts of C_2+_ products (FE_C2H4_ = 6.1%, FE_C2H5OH_ = 1.4%). Cu-1 shows similar product distribution as pristine Cu, suggesting that introduction of a thin film (*d* = 0.10 ± 0.01 µm) on the surface exerts no effect on CO_2_RR. With Cu-2 (*d* = 0.16 ± 0.02 µm), FE_CH4_ increases to 22.3%, while the improvement for C_2_H_4_ is marginal (FE_C2H4_ = 9.0%). In contrast, FE_C2H4_ increases to 16.2% on Cu-3 (*d* = 0.33 ± 0.05 µm) and 29.0% on Cu-4 (*d* = 0.62 ± 0.06 µm). It was desirable to test even thicker films−constructed by applying more negative potentials during grafting−but, unfortunately, no reliable results could be obtained because parts of the physically adsorbed film would break off during electrochemical deposition and electrolysis (Figs. S4 and S5).

In addition to selectivity, activity is a critical performance metric^38^, often measured by the turnover frequency. A straightforward approach to assess the activity consists of determining the relative values of the absolute partial current densities, normalized with respect to either the geometric area (|*j*_geo_|) or the electrochemical active surface area (|*j*_ECSA_|). Thus, we determined the ECSA of each individual electrode using the double-layer capacitance method prior to performing bulk electrolysis, while the partial current density of the individual product could be calculated based on the faradaic efficiencies (FEs) determined from product analysis (Fig. S6 and Table S3). Specifically, when layer thickness increases, ECSA and thus roughness factor (RF) decreases which we attribute to blocking of active sites by the nonconductive molecular film. For example, while RF = 2.5 ± 0.2 for pristine Cu, it is 1.9 ± 0.1 for Cu-5. In this regard, comparisons of intrinsic activity with |*j*_ECSA_| are therefore accurate within the variations of active site densities among samples. We also attempted to use the Pb underpotential deposition method to determine ECSAs^39,40^, but had to abandon it for the Cu-n electrodes because the presence of the organic films shifted the stripping curves in a positive potential direction to make it collide with the oxidation of bulk Cu itself (Fig. S7).

Figure 3b−d shows values of |*j*_geo_| and |*j*_ECSA_| obtained for CH_4_, C_2_H_4_, and H_2_ production at all Cu-n electrodes in CO_2_RR. Regarding CH_4_, the intrinsic activity in terms of |*j*_ECSA,CH4_| behaves as a volcano-like curve as function of *d* (Fig. 3b). Starting from |*j*_ECSA,CH4_| = 0.12 mA cm^−2^ on pristine Cu, it rises with increasing *d* until it passes a maximum of 0.24 mA cm^−2^ on Cu-2 (*d* = 0.16 ± 0.02 µm). From this point on, it decreases. In contrast, |*j*_ECSA,C2H4_| increases as function of *d* (Fig. 3c), with |*j*_ECSA,C2H4_| = 0.52 mA cm^−2^ on Cu-5 being 8.7 times higher than that on pristine Cu (|*j*_ECSA,C2H4_| = 0.06 mA cm^−2^). This means that |*j*_ECSA,C2H4_|/|*j*_ECSA,CH4_| increases from 0.4 to 18.2, with *d* going from 0.10 ± 0.01 µm for Cu-1 to 0.79 ± 0.08 µm for Cu-5. (Fig. S8). Similarly, |*j*_ECSA,C2+_| (0.68 mA cm^−2^) becomes 6.8 times larger than that on pristine Cu (0.10 mA cm^−2^) (Table S3). Thus, a thick and porous T-bipyridine film improves the activity for C_2_H_4_ while inhibiting CH_4_ production. Note that the total |*j*_ECSA_| is, by and large, independent of film thickness and, in fact, whether the film is there or not (Table S3).

The HER is slightly suppressed by the organic film, with |*j*_ECSA,H2_| declining from 0.51 mA cm^−2^ on pristine Cu to 0.28 mA cm^−2^ on Cu-5 under CO_2_ atmosphere (Fig. 3d). If the bulk electrolysis is performed under Ar atmosphere with no simultaneous CO_2_RR occurring, |*j*_ECSA,H2_| is found to be considerably larger in the range from 0.74−1.07 mA cm^−2^, independent of the film (Fig. 3e and Table S4). This suggests that in the absence of CO_2_, the diffusion rate of proton carriers, and through this HER, is unaffected by the presence of the organic film. However, with CO_2_ present HER becomes suppressed, most likely because of active sites being blocked by generated ^*^CO.

Previous work has reported that counter ions of the pyridinium salts, such as halide ions, could induce nanostructures on Cu surfaces^29,41^, potentially leading to improved performance for C_2+_ product formation during CO_2_RR. To rule out that the triflate anion in our case might corrode the Cu surface, we measured the root mean square roughness (*R*_q_) by AFM for a Cu surface, upon removal of the physisorbed T-bipyridine film by rinsing with acetone; the surface looks flat and smooth with *R*_q_ = 1.79 nm (Fig. S9). In comparison, *R*_q_ of pristine Cu is 1.07 nm (Fig. S9). This result is consistent with the SEM observations, i.e., no nanostructured Cu is visible on the surface (Fig. S10).

Next, X-ray photoelectron spectroscopy (XPS) measurements were conducted on pristine Cu and Cu-5 (Fig. S11) to reveal the extent to which the chemical state of Cu electrode would be altered by introducing the T-bipyridine film. As seen, two binding energy peaks at 932.4 and 952.1 eV are observed on both electrodes, attributed to the Cu 2*p* spectra of Cu 2*p*_3/2_ and Cu 2*p*_1/2_ signals, respectively^42^. Lack of large shakeup satellite peaks from 938−946 eV is consistent with the absence of Cu^2+^ species. Usually, it is difficult to distinguish Cu^0^ from Cu^+^ because of a too-small difference of the binding energies. However, the Cu LMM Auger spectra with kinetic energy peaks at 916.8 and 918.6 eV confirm the presence of Cu^+^ as well as Cu^0^ on both pristine Cu and Cu-5^43^. Thus, the formation of a native Cu_2_O layer cannot be avoided after exposure to ambient air. Still, the main conclusion is that no noteworthy valence change is found between pristine Cu and Cu-5, showing that the chemical state of the surface Cu is left unchanged by the T-bipyridine film.

### Thickness

**Fig. 4:**
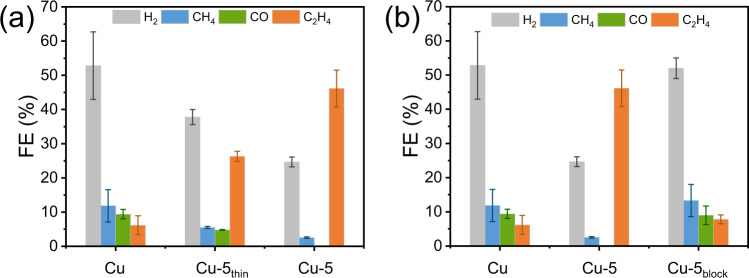
Effect of film thickness and porosity. **a** FEs for H_2_ (gray), CH_4_ (blue), CO (green), and C_2_H_4_ (orange) production at pristine Cu, Cu-5_thin_, and Cu-5 electrodes. **b** FEs for H_2_ (gray), CH_4_ (blue), CO (green), and C_2_H_4_ (orange) production at pristine Cu, Cu-5, and Cu-5_block_ electrodes. All bulk electrolysis experiments were conducted at an applied potential of −0.96 V versus RHE for 60 min in CO_2_-saturated 0.1 M KHCO_3_. Error bars correspond to the standard deviation of at least two independent measurements. Source data are provided as a Source Data file.

Already it has been shown that a thick and porous T-bipyridine film improves the activity of C_2_H_4_ while inhibiting CH_4_ production. To test the effect of *d* while keeping porosity intact, we prepared a variant of the Cu-5 sample (denoted Cu-5_thin_) using a deposition duration period of 300 s instead of 3600 s. SEM pictures validate the morphological similarity of Cu-5_thin_ and Cu-5 (Fig. S12), while the average *d* of the former, as expected, is significantly lower (0.15 ± 0.03 µm versus 0.79 ± 0.08 μm) according to AFM measurements (Fig. S13). The product distribution in CO_2_RR shows a noticeable change (Fig. 4a), with FE_C2H4_ declining from 46.1 to 26.3%, FE_C2+_ decreasing from 61.9 to 34.7% (Table S5), and FE_CH4_ increasing from 2.5 to 5.5% going from Cu-5 to Cu-5_thin_. A plausible explanation of this finding is that the residence period of in situ generated ^*^CO on the surface decreases with the decrease of *d*, resulting in a poorer conversion of CO_2_ to C_2+_ products. A major aim of future work is to test this effect of *d* by creating thicker organic films than the already achieved 0.79 μm, if the stability issue of the physically adsorbed organic films can be solved.

### Porosity

SEM pictures demonstrate the porous structure of the T-bipyridine film once the applied potential becomes more negative than −1.0 V versus RHE (Fig. 2b–f). Formation of gaseous products on the electrode surface under these conditions, originating from both HER and CO_2_RR, will create cavities and voids in the organic film during the deposition process^44^. To shed light on the porosity effect in a direct, but also destructive way, the organic film on the best-performing electrode, Cu-5 was exposed to 50 µL acetone, drop cast directly onto the organic film. This dissolved the film which was then allowed to slowly rebuild upon acetone evaporation. As seen, the resulting organic film is characterized by a lamellar and nonporous structure, denoted Cu-5_block_ (Fig. S14). Notably, the thickness (*d* = 0.13 ± 0.02 µm; Fig. S15) is lower than that of Cu-5 (*d* = 0.79 ± 0.08 µm) which can be converted to a value of *P* = 83% for Cu-5 (see Table S2). In comparison, the corresponding *P*´s for Cu-1, Cu-2, Cu-3, and Cu-4 are 55%, 50%, 67% and 80%, respectively.

The CO_2_RR performance of Cu-5_block_ was evaluated under identical conditions as for Cu-5 (Fig. 4b and Table S6). Strikingly, the selectivity for both C_2_H_4_ (FE_C2H4_ = 7.8%) and C_2+_ products (FE_C2+_ < 10.0%) is considerably lower for Cu-5_block_, with |*j*_ECSA__-__C2H4_| being 5.7 times smaller than that of Cu-5 (Table S6). Importantly, this cannot be attributed to the change in film thickness caused by film restructuring. Although the film on Cu-5_block_ is small in size, becoming close to that of Cu-5_thin_ (0.15 µm), its performance is inferior to that of the latter. This demonstrates the significance of the organic film structure in CO_2_RR, with the hypothesis that a high-porosity film structure can secure optimal conditions for C_2+_ product formation by speeding up CO_2_ diffusion and retaining in situ generated ^*^CO (higher partial pressure).

### Mass transport

In previous work on CO_2_RR, the porosity of the polymer was demonstrated to be important for mass transport of the species concerned^31,45^. In fact, the specific morphology of the polymer could even facilitate the supply of CO_2_ to the Cu-polymer interface by speeding up the diffusion of CO_2_^46^. To examine the deposited films from this perspective, we selected a well-known redox probe, 1,1′-dimethyl-4,4′-bipyridinium dichloride (methyl viologen), for a cyclic voltammetric study. In addition to exhibiting a profound redox wave in a convenient potential range, the relatively large molecular size and double-charged nature of this probe can be used to put the transport capabilities of the film to the test.

Cyclic voltammetry of methyl viologen was first performed on pristine Cu using various sweep rates (*v*’s) from 0.5−2.0 V s^−1^ in Ar-saturated 0.1 M KHCO_3_ (Supplementary Note 1 and Fig. S16). The peak current increases with *v*^1/2^ which is in line with a diffusion-controlled process^47^. Next, voltammograms were recorded on the Cu-5 electrode, with the redox wave now showing an S-shaped feature and being slightly reduced in size (Fig. S17). This suggests a change in the diffusion mode, going from planar to spherical^47^, which, typically, is seen for ultra-microelectrodes or arrays of such. This would infer that the structure of the organic film images an ultra-microelectrode array quite well by prohibiting methyl viologen from getting through all pore sites in the film. Considering the quite large molecular size and double-charged nature of methyl viologen, this is not surprising. In a sense, it is more surprising that the film is porous to an extent that allows a significant penetration of this probe. It would suggest that the smaller CO_2_ and H_2_O molecules should be in an excellent position to gain access to the Cu surface.

Along these lines, the hydrophobicity of the organic films should be considered, also because other studies have demonstrated that submerged hydrophobic surfaces can trap gas at the nanoscale, thereby accumulating CO_2_ and repelling isolated H_2_O at the electrode-electrolyte interface^48,49^. To assess the hydrophobicity of the deposited T-bipyridine film, water contact angle measurements were performed (Fig. S18). As expected, the pristine Cu electrode is hydrophilic with a contact angle of 22°. In comparison, the surfaces of Cu-n (n = 1–5) are considerably more hydrophobic, with water contact angles ranging between 75° and 115°. Nevertheless, the performance of the pristine Cu and Cu-1 electrodes are almost identical, despite the different water contact angles measured for these two cases. This would imply that with thickness changes, issues related to hydrophobicity/hydrophilicity are not as important as the relative diffusion (and thus reaction rate) changes pertaining to CO_2_, CO, or H_2_O caused by the porosity effect. In our study, the HER performance in absolute terms under Ar atmosphere is unaffected by the T-bipyridine film (Fig. 3e and Table S4), showing a limited effect from film hydrophobicity. Besides the high porosity of the film, a possible reason for this is the applied electric field during electrolysis that can induce wetting of the hydrophobic film, thus making it more accessible for H_2_O^50^. In conclusion, the T-bipyridine films seem to impose no severe restrictions on the diffusion process of neither CO_2_ nor H_2_O. As a final point, the electrolyte composition (in particular, the cation) would be expected to exert a great impact on the reaction performance^23^. This aspect was not studied further, being outside the scope of the present study.

### Mechanistic insights from *operando* Raman spectroscopy

To gain insight into the C–C coupling mechanism in the presence of the T-bipyridine film, *operando* Raman spectroscopy measurements were performed on both pristine Cu and Cu-5 electrodes. At open circuit potential, multiple bands at 427, 528, and 616 cm^−1^ appear (Fig. S19). These are attributed to Cu_2_O^51^ in line with formation of a native oxide layer on Cu after exposure to ambient air. The intensity of the Cu_2_O bands decreases (from 0 to 20 s) and finally vanishes (25 s) upon applying a negative potential of −0.2 V versus RHE, as this causes reduction of the oxide layer (Fig. S20). Accordingly, catalytic sites consist of metallic Cu rather than Cu_2_O, in accordance with earlier reports^52,53^.

**Fig. 5:**
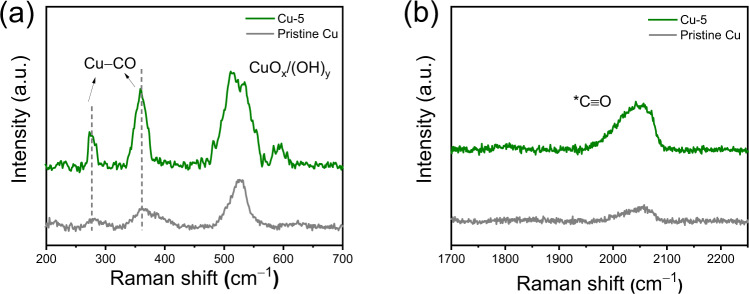
*Operando* Raman spectroscopy. Spectra were collected for pristine Cu (gray) and Cu-5 (green) during CO_2_ reduction at two regions, i.e., **a** low wavenumber range at 200–700 cm^−1^ and **b** high wavenumber range at 1700–2250 cm^−1^ at an applied potential of −0.9 V versus RHE in CO_2_-saturated 0.1 M KHCO_3_. Source data are provided as a Source Data file.

Figure 5a shows two bands appearing at 280 and 366 cm^−1^ at a potential of −0.9 V versus RHE in CO_2_-saturated 0.1 M KHCO_3_, corresponding to the frustrated *ρ* (Cu–CO) rotational mode and *v* (Cu–CO) stretching mode, respectively^54^. The band between 1950 and 2100 cm^−1^ arises from C$\equiv$O stretching of the ^*^CO adsorbed on metal sites (Fig. 5b)^55,56^. Notably, the intensity of these bands is greater on Cu-5 than pristine Cu, suggesting that the presence of the T-bipyridine film enhances ^*^CO coverage which will better enable C_2+_ product formation through C–C coupling. Moreover, a blue shift of the CO stretching band would be anticipated if CO is bound strongly to Cu^22^, but we note that the Cu–CO stretching band is located in the same position on Cu-5 as on pristine Cu, implying that the Cu–CO bond strength is identical in the two cases. This also shows that the film itself is incapable of stabilizing the ^*^CO intermediate to an extent where this comes through as a decrease of the adsorption energy. Thus, the enhanced CO_2_RR activity cannot be attributed to changed energetics of ^*^CO, but instead to a larger coverage resulting from higher local CO partial pressure.

### Microkinetic model

To corroborate our working hypothesis on the key role of the partial pressure of CO in the organic films, we developed a microkinetic model to depict the alterations in selectivity on the Cu surface (see Supplementary Note 2 in the Supporting Information). The goal is to build a basic model with as few assumptions as possible that would still be capable of qualitatively characterizing the change in product distribution with varying amounts of carbon reactants (e.g., CO) and hydrogen reactants (e.g., protons) at the surface. This microkinetic model is derived from the Langmuir adsorption expressions as shown in Eqs. (2)−(4).

2 $${\theta }_{\ast }=\frac{1}{1+{K}_{{{{{{\rm{A}}}}}}}{P}_{{{{{{\rm{A}}}}}}}+{K}_{{{{{{\rm{B}}}}}}}{P}_{{{{{{\rm{B}}}}}}}}$$

3 $${\theta }_{{{{{{\rm{A}}}}}}}=\frac{{K}_{{{{{{\rm{A}}}}}}}{P}_{{{{{{\rm{A}}}}}}}}{1+{K}_{{{{{{\rm{A}}}}}}}{P}_{{{{{{\rm{A}}}}}}}+{K}_{{{{{{\rm{B}}}}}}}{P}_{{{{{{\rm{B}}}}}}}}$$

4 $${\theta }_{{{{{{\rm{B}}}}}}}=\frac{{K}_{{{{{{\rm{B}}}}}}}{P}_{{{{{{\rm{B}}}}}}}}{1+{K}_{{{{{{\rm{A}}}}}}}{P}_{{{{{{\rm{A}}}}}}}+{K}_{{{{{{\rm{B}}}}}}}{P}_{{{{{{\rm{B}}}}}}}}$$

Here, A and B represent two different adsorbates, corresponding to hydrogen (H) and carbon (C) in our system; ${\theta }_{*}$, ${\theta }_{{{{{{\rm{A}}}}}}}$, and ${\theta }_{{{{{{\rm{B}}}}}}}$ are the coverages on the surface of free sites, adsorbate A, and adsorbate B, respectively; ${K}_{{{{{{\rm{A}}}}}}}$ and ${K}_{{{{{{\rm{B}}}}}}}$ are the equilibrium constants of adsorbates A and B, respectively; and $P$ is the partial pressure of the given adsorbate.

The Langmuir adsorption model has the following inherent assumptions: 1) equilibrium is ensured, i.e., the chemical potential of the adsorbate is the same in gas phase as on the surface, 2) one monolayer of adsorbate at most is attainable, 3) adsorbate–adsorbate interactions are ignored, and 4) surface sites are all equivalent. The FEs for the various product are calculated from the rate equations for production of H_2_, C_1_ (CH_4_, CO, and HCOO^−^), C_2_ (CH_3_COO^−^, C_2_H_4_, and C_2_H_5_OH), and C_3_ (C_3_H_7_OH) products.

The rate equations are given by Eqs. (5)−(8).

5 $${r}_{{{{{{\rm{C}}}}}}1}={k}_{{{{{{\rm{C}}}}}}1}{P}_{{{{{{\rm{C}}}}}}}{P}_{{{{{{\rm{H}}}}}}}^{O({{{{{\rm{H}}}}}},a)}{\theta }_{\ast }^{O(\theta,a)}$$

6 $${r}_{{{{{{\rm{C}}}}}}2}={k}_{{{{{{\rm{C}}}}}}2}{P}_{{{{{{\rm{C}}}}}}}^{2}{P}_{{{{{{\rm{H}}}}}}}^{O({{{{{\rm{H}}}}}},b)}{\theta }_{\ast }^{O(\theta,b)}$$

7 $${r}_{{{{{{\rm{C}}}}}}3}={k}_{{{{{{\rm{C}}}}}}3}{P}_{{{{{{\rm{C}}}}}}}^{3}{P}_{{{{{{\rm{H}}}}}}}^{O({{{{{\rm{H}}}}}},c)}{\theta }_{\ast }^{O(\theta,c)}$$

8 $${r}_{{{{{{\rm{H}}}}}}2}={k}_{{{{{{\rm{H}}}}}}2}{P}_{{{{{{\rm{H}}}}}}}^{O({{{{{\rm{H}}}}}},d)}{\theta }_{\ast }^{O(\theta,d)}$$

Here, *r* is the reaction rate, *k* is the forward rate constant, *P* is the partial pressure, ${\theta }_{*}$ is the coverage of free sites, and *O* is the reaction order for either H or ${\theta }_{*}$, with *a*, *b*, *c*, and *d* indicating the corresponding reaction order for the formation of (a) C_1_, (b) C_2_, (c) C_3_, and (d) H_2_.

**Fig. 6:**
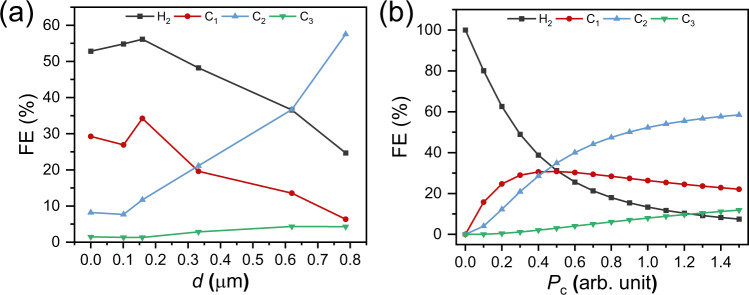
Microkinetic model. Comparison of FEs of H_2_ (black), C_1_ (red), C_2_ (blue), and C_3_ (green) from **a** experimental results as function of *d* and **b** model results as function of carbon partial pressure *P*_C_ (arb. unit) at the surface. Source data are provided as a Source Data file.

Figure 6 shows FEs for H_2_, C_1_, C_2_, and C_3_ products for modified Cu as function of *d* (i.e., thickness) for the experimental results (Fig. 6a), and as function of carbon partial pressure (*P*_C_) at the surface for the microkinetic model (Fig. 6b). Experimentally, both FE_C2_ and FE_C3_ increase while FE_H2_ decreases with *d*. Interestingly, FE_C1_ exhibits volcano-like behavior with an increase of *d* until 0.16 ± 0.02 μm, followed by a steady decrease. Encouragingly, the microkinetic model shows the exact same trend for all products, with *P*_C_ as the primary descriptor. While we have not established a direct relationship between *d* and *P*_C_ on the Cu surface, we see similar qualitative trend in the experimental *d*´s at 0–0.79 µm and the model *P*_C_ data at 0.3–1.0. This backs up the findings from the experiments, corroborating the hypothesis that the molecular film enhances ^*^CO coverage at the surface.

Previously, analyzes of CO_2_ and H_2_O diffusion coefficients in water and various polymeric systems pointed in the same direction^32,33,57^. Christensen et al. discovered that the molecular modifier facilitated the diffusion of CO_2_ relative to H_2_O^57^. It was hypothesized that this would alter the chemical potential (and concentration) at the surface in favor of CO_2_ and CO, increasing the C–C coupling required for C_2+_ products, while lowering HER selectivity. Fig. S21 displays the model findings for greater values of *P*_C_ and proton partial pressure (*P*_H_), demonstrating that the model has the expected asymptotic behavior at high *P*_H_, with H_2_ generation dominating when *P*_H_ >> *P*_C_.

This research demonstrates that the activity and selectivity of Cu for CO_2_RR can be modulated by adjusting the physical structure of a deposited organic film, with emphasis on porosity and thickness. With a total FE of 61.2%, the best-performing electrode shows strong CO_2_RR activity toward C_2+_ products. Although essentially no changes are observed to the total activity, once normalizing the current density with the ECSA of each electrode, the intrinsic activity of C_2_H_4_ improves 8.7-fold compared with an analogous polycrystalline Cu electrode with no active layer. AFM, SEM, and XPS studies rule out noticeable contributions from surface nanostructuring or oxidation state changes. Instead, mechanistic insights from *operando* Raman measurements and a microkinetic model indicate that the greater local CO partial pressure and ^*^CO surface coverage are responsible for the activity and selectivity changes. On a ^*^CO intermediate-rich Cu surface, C–C coupling would be enhanced, thus altering the reaction pathway from C_1_ to C_2+_ products. Overall, this study proposes an approach for enhancing the CO_2_-to-C_2+_ conversion by customizing the physical structure of the modifier and the microenvironment at the electrode–electrolyte interface.

## Methods

### Materials

All solvents and reagents were obtained from commercial sources (Aldrich and Merck) and used as received, unless stated otherwise. Cu foil (99.99%) was purchased from Goodfellow. Deuterium dioxide (D 99.96%), d-chloroform (D 99.8%), and d6-dimethylsulfoxide (D 99.8%) were purchased from Cambridge Isotope Laboratories. Water was purified by a Nanopure Analytical Ultrapure Water System (Thermo Scientific) or a Milli-Q Advantage A10 Water Purification System (Millipore) with specific resistance of 18.2 MΩ cm at 25 °C. ^1^H nuclear magnetic resonance (NMR) spectra were recorded on a Bruker 400 MHz instrument with a prodigy broadband cryoprobe. Shifts were reported relative to the residual solvent peak.

### Characterization of electrodes

XPS spectra were recorded on a Kratos Axis Ultra-DLD instrument using monochromatic Al Kα X-ray source at 150 W. Measurements were performed while keeping pressure below 5 × 10^−12^ bar. Full survey spectra were acquired with pass energy of 160 eV and high-resolution spectra of C 1*s*, N 1*s*, Cu 2*p *and Cu LMM with 20 eV. CasaXPS software was employed for deconvolution and analysis of spectra. SEM images were acquired on a FEI Magellan 400 SEM instrument equipped with a through-the-lens detector at 5 kV accelerating voltage. All AFM images were captured by commercial Dimension Icon AFM (Bruker, Santa Barbara, USA). Topographies of samples were imaged in the tapping mode with ultrasharp probes (RTESP-37) in ambient pressure condition. The water contact angle was measured by the sessile drop method using an FTA1000 contact angle system (First Ten Angstroms, Inc.) at ambient temperature. The volume of the water droplets was 2.0 µL.

### Electrochemical analysis

Linear sweep voltammetry and chronoamperometry measurements were carried out in a custom-made PEEK flow cell setup similar to the one reported by ref. ^6^, with two chambers separated by Sustainion® X37–50 exchange membrane. The three-electrode system consisted of pristine Cu or T-bipyridine modified Cu foil as working electrode, a platinum foil as counter electrode, and leak-free Ag/AgCl (Innovative Instruments Inc.) as reference electrode. O-rings were used to secure the working and counter electrodes, with exposed surface area of 1.22 cm^2^ on each. Supporting electrolyte (2 mL for each chamber) was either Ar-saturated 0.1 M KHCO_3_ (pH = 8.4) or CO_2_-saturated 0.1 M KHCO_3_ (pH = 6.8). All electrochemical measurements were carried out using an Autolab potentiostat (PGSTAT204). Potentiostatic electrochemical impedance spectroscopy (PEIS) using frequencies from 0.1 to 100 kHz was carried out prior to each electrolysis experiment to determine ohmic resistance of the flow cell. Typically, resistances were in a range from 65 to 75 Ω.

The applied potential measured against Ag/AgCl was converted to the RHE with *iR* correction (*i* is the current and *R* is the resistance measured by PEIS) using Eq. (9).

9 $$E({{{{{\rm{versus}}}}}}\,{{{{{\rm{RHE}}}}}})=E({{{{{\rm{versus}}}}}}\,{{{{{\rm{Ag}}}}}}/{{{{{\rm{AgCl}}}}}})+0.197+0.059\,{{{{{\rm{pH}}}}}}-iR$$

Bulk electrolysis was performed at an applied potential of −0.96 V versus RHE with CO_2_ or Ar gas stream flowing into the cell (2.3 mL min^−1^) from the bottom for 60 min. Gaseous products (H_2_, CO, CH_4_, and C_2_H_4_) were quantified by an Agilent Technologies 7890B gas chromatography system equipped with a thermal conductivity detector and flame ionization detector. Every 15 min, 250 µL of gas was sampled to determine the concentration of gaseous products. FEs of products were calculated as the average of four separate injections. After electrolysis, 1 mL of catholyte was mixed with 200 µL deuteroxide and 70 µL of 10 mM DMSO/D_2_O (internal standard) in a glass sample tube. Half of the mixture (635 µL) was transferred to a NMR tube and analyzed by ^1^H NMR spectroscopy to quantify liquid products by comparing the ^1^H peak area of each product with that of DMSO. The signal of water was suppressed using a presaturation sequence.

The partial current density of a specific product (|*j*|) normalized with respect to the geometric surface area (*A*_geo_ = 1.22 cm^2^) and ECSA (*A*_ECSA_) of the electrode was obtained by Eqs. (10) and (11).

10 $$|{j}_{{{{{{\rm{geo}}}}}}}|=\frac{I\,({{{{{\rm{average}}}}}}\,{{{{{\rm{current}}}}}})\times {{{{{\rm{FE}}}}}}\,(\%)}{{A}_{{{{{{\rm{geo}}}}}}}}$$

11 $$|{j}_{{{{{{\rm{ECSA}}}}}}}|=\frac{I\,({{{{{\rm{average}}}}}}\,{{{{{\rm{current}}}}}})\times {{{{{\rm{FE}}}}}}\,(\%)}{{A}_{{{{{{\rm{ECSA}}}}}}}}$$

In the equation, *I* represents the absolute average current during electrolysis.

### Electrode preparation

Polycrystalline Cu foil (99.99% metal basis, 1 mm thickness, Goodfellow) was cut into 2.5 × 2.5 cm^2^ square pieces and polished with sandpaper and diamond slurries (9, 3, 1, and 0.25 µm). Subsequently, these cut-out electrodes were electrochemically polished in 85 wt% phosphoric acid by applying a potential of 2.1 V versus a graphite rod counter electrode for 5 min to remove residuals from the diamond slurries. Finally, they were rinsed with Milli-Q water and dried in a stream of Ar.

T-bipyridine film was obtained from electroreductive dimerization of 10 mM 1-(4-tolyl)pyridinium triflate (T-Pyr), with synthesis details of T-Pyr provided in Supplementary Notes 3 and 4, and Figs. S22 and S23. Electrochemical film assembling took place on the polycrystalline Cu electrodes in CO_2_-saturated 0.1 M KHCO_3_ (pH = 6.8) using the same setup as for bulk electrolysis. Film thickness and porosity could be controlled by applying a potential ranging from −1.25 to −0.55 V versus RHE, with the latter potential determined from cyclic voltammetry recording (Fig. S24). Deposition conditions are summarized in Table S1.

### Electrochemical active surface area (ECSA) and roughness factor (RF)

ECSA and RF of each single electrode were determined by measuring the double-layer capacitance (*C*_DL_) of the system prior to bulk electrolysis. First, a non-Faradaic potential range was identified to be from −0.05 to 0.05 V versus RHE from cyclic voltammetry in quiescent solution. Then cyclic voltammetry was performed at *v*’s from 20 to 120 mV s^−1^ in CO_2_-saturated 0.1 M KHCO_3_ (pH = 6.8). The absolute electrochemical double-layer capacitive current density, |*j*_c_|, was calculated by averaging the cathodic and anodic current densities at 0 V versus RHE from the cyclic voltammograms. The value of *C*_DL_ was calculated by plotting |*j*_c_| against *v*, noting that ECSA is proportional to *C*_DL_ (Fig. S6)^58,59^. With this, RF of the Cu-based catalyst could be calculated as *C*_DL_/*C*_S_ where *C*_S_ is the specific capacitance of the material; we use *C*_S_ = 29 µF cm^−2^ for electropolished polycrystalline Cu foil^60^. Current densities recorded were normalized with respect to either geometric area (|*j*_geo_| using *A*_geo_ = 1.22 cm^2^) or ECSA (|*j*_ECSA_| using the measured RF × *A*_geo_ as *A*_ECSA_).

### *Operando* Raman spectroscopy

A three-electrode electrochemical cell with a quartz window by E-CATA (Shen Zhen) Instrument Equipment Company was used for *operando* Raman measurements (Fig. S25). Graphite and leak-free Ag/AgCl served as counter and reference electrode, respectively. Pristine Cu foil or Cu-5 electrode was used as working electrode. The counter electrode was separated from the working electrode by a Sustainion® X37–50 exchange membrane to avoid cross-contamination. Electrolyte (CO_2_-saturated 0.1 M KHCO_3_) was forced to flow into the cell by a peristaltic pump to replenish CO_2_ and remove generated gas bubbles, which would interfere with the Raman signal collection. All electrochemical tests were conducted using a CHI potentiostat. *Operando* Raman spectra were recorded versus time employing a Renishaw inVia Raman spectrometer and a near-infrared laser (785 nm) excitation source. Each spectrum was the representative of multiple measurements at different time scale of electrolysis. Signal acquisition time for each Raman spectrum was ~50 s (20 accumulations).

## Supplementary information

Supplementary Information

## Data Availability

Synthetic procedures, NMR spectra as well as electrochemical and characterization data are available in the Supplementary Information. Source data are provided with this paper.

## Source data

Source Data

## Code availability

The script for the microkinetic model is available at https://sid.erda.dk/cgi-sid/ls.py?share_id=e3OC2vAbe1.
