# Supplementary material for: Steering carbon dioxide reduction toward C–C coupling using copper electrodes modified with porous molecular films
Source: Nat Commun. 2023 Feb 15;14:844. doi: 10.1038/s41467-023-36530-z (PMC9932156; doi:10.1038/s41467-023-36530-z)
Supplement: Supplementary file 1 — Supplementary Information [file 41467_2023_36530_MOESM1_ESM.pdf]

SUPPORTING INFORMATION

Steering Carbon Dioxide Reduction Toward C–C Coupling Using Copper Electrodes Modified with Porous Molecular Films

Siqi Zhao[1,2], Oliver Christensen[3], Zhaozong Sun[1], Hongqing Liang[4], Alexander Bagger[3], Kristian Torbensen[1,2], Pegah Nazari[2,5], Jeppe Vang Lauritsen[1], Steen Uttrup Pedersen[1,5], Jan Rossmeisl[3,*] and Kim Daasbjerg[1,2,5,*]

[1]Interdisciplinary Nanoscience Center (iNANO), Gustav Wieds Vej 14, DK-8000 Aarhus C, Denmark
[2]Novo Nordisk Foundation (NNF) CO_2 Research Center, Aarhus University, Gustav Wieds Vej 10C, 8000 Aarhus, Denmark
[3]Department of Chemistry, University of Copenhagen, Universitetsparken 5, Copenhagen, Denmark
[4]Leibniz-Institut für Katalyse, Albert-Einstein-Straße 29a, 18059 Rostock, Germany

[5]Department of Chemistry, Langelandsgade 140, 8000 Aarhus, Denmark

[*]Correspondence to: jan.rossmeisl@chem.ku.dk (J. R.); kdaa@chem.au.dk (K. D.).

Table of Contents

Supplementary Notes

Diffusion behavior of methyl viologen in T-bipyridine film S3
Microkinetic model S4
Synthesis S5
Quantification of para/ortho ratios in T-bipyridine film S6

Supplementary Figures

SEM images of pristine Cu and Cu-1–Cu-5 prior to CO_2RR S7
SEM images of electrografted Cu/T-bipyridine film electrode S8
Optical micrographs of electrografted Cu/T-bipyridine film electrode S9
ECSA measurements...S10
Pb underpotential deposition (Pb UPD) S11
Partial current density ratio of C_2H_4/CH_4...S12
AFM images of pristine Cu and Cu-5 after removal of electrografted T-bipyridine film S13
SEM images of pristine Cu and Cu-5 after removal of electrografted T-bipyridine film S14
XPS spectra of pristine Cu and Cu-5 prior to CO_2RR S15
SEM images of Cu-5_{thin} prior to CO_2RR...S16
AFM image of Cu-5_{thin} prior to CO_2RR..S17
SEM images of Cu-5_{block} prior to CO_2RR...S18
AFM image of Cu-5_{block} prior to CO_2RR..S19
Cyclic voltammogram of methyl viologen on pristine Cu..............................S20
Cyclic voltammogram of methyl viologen on pristine Cu and Cu-5 ....S21
Water contact angle for pristine Cu and Cu-n...S22
Operando Raman spectra of Cu and Cu-5 recorded at OCP S23
Operando Raman spectra of Cu and Cu-5 recorded at −0.2 V versus RHE....S24
Microkinetic model for greater P_C and P_H..S25
^{1}H NMR spectra of synthesized molecules...S26
^{1}H NMR spectrum of dimer molecule obtained after electrodimerization of 1-(4-tolyl)pyridinium triflate salt S27
Cyclic voltammogram of 1-(4-tolyl)pyridinium triflate salt...........................S28
Operando Raman electrochemical cell S29

Supplementary Tables

Table of deposition conditions of Cu-n...S30
Table of film porosity of Cu-n S31
Table of CO_2RR performance on pristine Cu and Cu-n S32
Table of HER performance on pristine Cu and Cu-n.......................................S34
Table of CO_2RR performance on Cu-5_{thin}...S35
Table of CO_2RR performance on Cu-5_{block}...S36
Supplementary References...S37

Supplementary Note 1

Diffusion behavior of methyl viologen in T-bipyridine film

Cyclic voltammetry of 2 mM 1,1'-dimethyl-4,4'-bipyridinium dichloride (i.e. methyl viologen) was first recorded on pristine Cu (area = 0.0314 cm^2) using various sweep rates (v's) from 0.5–2.0 V s^{-1} in Ar-saturated 0.1 M $KHCO_3$ (pH = 8.4; Fig. S16a,b). Fig. S16c,d demonstrates that the absolute peak current density times $v^{-1/2}$, i.e. $|j_p|v^{-1/2}$, is constant. This is in accordance with the prediction of the Randles-Sevcik equation [equation (S1)].[1]

$$i_p = 0.4463nFAC\sqrt{\frac{nFvD}{RT}} \tag{S1}$$

Here, i_p = peak current, n = number of electrons transferred in the redox event, A = electrode area, F = Faraday's constant, D = diffusion coefficient, C = concentration, R = gas constant, and T = temperature.

Next, cyclic voltammetry of methyl viologen was recorded on Cu-5 (area = 0.0314 cm^2) using v = 50 mV s^{-1} in Ar-saturated 0.1 M $KHCO_3$ (pH = 8.4; Fig. S17).

Supplementary Note 2

Microkinetic model

The full script for the microkinetic model, along with additional figures and videos showing results for the model, is available here.

The basis for the model is the rate equations for production of H_2, C_1 (CH_4, CO and $HCOO^-$), C_2 (CH_3COO^-, C_2H_4 and C_2H_5OH), and C_3 (C_3H_7OH) products. For simplicity's sake, we only consider the partial pressures of carbon (P_C) and hydrogen (P_H), despite oxygen (O) also being involved in the reaction. In addition, the carbon (C) corresponds to the amount of carbon-containing reactants (e.g. CO) at the surface and the hydrogen (H) corresponds to the amount of hydrogen-containing reactants (e.g. proton) at the surface.

Qualitatively, this is similar to the Anderson-Flory-Schulz model that the Fischer-Tropsch process follows for the product distribution of hydrocarbons,[2] although that model also takes into account products from the competing HER, along with the relative pressure of C compared to H at the surface. We only consider carbon chains up to C_3, as the selectivity for longer chains is insignificant for standard CO_2 reduction on Cu in aqueous solution.

The Faradaic efficiency (FE) for a given product X is calculated by equation (S2).

$$\mathrm{FE_X} = \frac{r_\mathrm{X}}{r_\mathrm{C1}+r_\mathrm{C2}+r_\mathrm{C3}+r_\mathrm{H2}} = \frac{|j_\mathrm{X}|}{|j_\mathrm{C1}|+|j_\mathrm{C2}|+|j_\mathrm{C3}|+|j_\mathrm{H2}|} \quad \text{(S2)}$$

Note that the rate r and the absolute current density $|j|$ are considered equivalent for our purposes.

Reaction orders of H and θ_*, O(H) and $O(\theta_*)$ respectively, for the four different reactions, are given as follows:

$$O(\mathrm{H,a}) = 1, O(\theta,a) = 1 \ (\mathrm{C_1})$$

$$O(\mathrm{H,b}) = 1, O(\theta,b) = 2 \ (\mathrm{C_2})$$

$$O(\mathrm{H,c}) = 1, \ O(\theta,c) = 3 \ (\mathrm{C_3})$$

$$O(H,\mathrm{d}) = 2, O(\theta,d) = 1 \ (\mathrm{H_2})$$

The values are chosen based on the assumption that (a) increasingly long carbon chains should require a correspondingly higher number of free sites for C–C coupling to occur and (b) H_2 production should be more dependent on H partial pressure than CO_2R products as H_2 production would, otherwise, never dominate. These parameters are chosen to qualitatively correspond to experimental trends, rather than to quantitatively recreate experimental results.

The rate constants are obtained by fitting the model to CO_2RR data from Fig. 5 and 6 in Kuhl et al.[S3] at a single point (–1.05 V versus RHE corresponding to starting parameters $P_C = 1.0$ and $P_H = 2.0$ in our model), then solving a set of four equations with four unknowns.

Supplementary Note 3

Synthesis

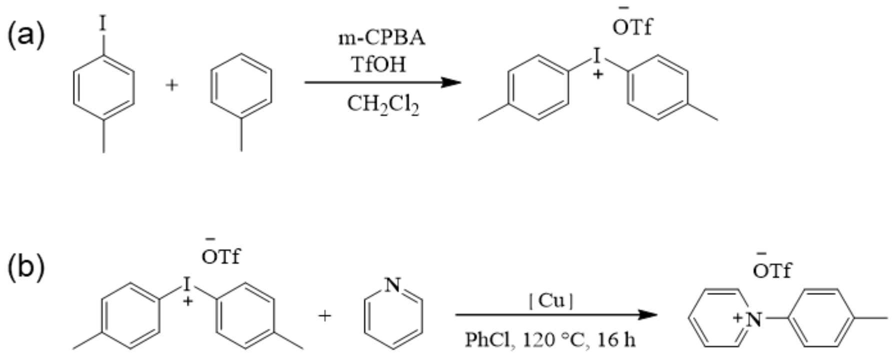

Supplementary Fig. S1. Synthesis of (a) bis(4-tolyl)iodonium triflate salt and (b) 1-(4-tolyl)pyridinium triflate salt.

Bis(4-tolyl)iodonium triflate was synthesized according to a reported procedure (Fig. S1a).[4,5] 4-Iodotoluene (1.09 g, 5.0 mmol, 1 equiv.), toluene (0.6 mL, 5.5 mmol, 1.1 equiv.), meta-chloroperbenzoic acid (1.39 g, 5 mmol, 1.1 equiv.) were dissolved in 25 mL CH_2Cl_2 in a round-bottom flask equipped with a magnetic stir bar. Trifluoromethanesulfonic acid, TfOH (0.88 mL, 10.5 mmol, 2.1 equiv.), was added dropwise yielding a dark brown solution. The solution was concentrated under reduced pressure before adding 25 mL diethyl ether. The desired product precipitated as white powder and was collected by filtration. The powder was washed with cold diethyl ether (5 × 20 mL) and dried under vacuum. Yield: 53%.

1-(4-Tolyl)pyridinium triflate salt was synthesized following the procedure in Fig. S1b.[1,2] Bis(4-tolyl)iodonium triflate (916.5 mg, 2.0 mmol, 1 equiv.), copper(II) stearate (37.8 mg, 0.06 mmol, 0.03 equiv.), and pyridine (0.16 mL, 2.0 mmol, 1 equiv.) were suspended in 5 mL chlorobenzene (PhCl) under Ar atmosphere in a Schleck round-bottom flask equipped with a magnetic stir bar. The mixture was heated at 110 °C for 16 h. The solution was cooled down to ambient temperature before adding diethyl ether (20 mL). The precipitate formed was isolated by filtration and washed with cold diethyl ether (5 × 20 mL). The product was purified further by recrystallizing from methanol/diethyl ether and stored in fridge (4 °C) over 5 h. The final product was collected by filtration and dried under vacuum. Yield: 69%.

Supplementary Note 4

Quantification of para/ortho ratios in T-bipyridine film

Previously, tetrahydrobipyridine was characterized spectroscopically, showing that coupling in both para and ortho positions is possible for the molecule utilized in this work.[4,5] In fact, the ^{1}H NMR spectrum (Fig. S23) indicates that the film extracted from the copper electrode is principally made of para-para and ortho-ortho dimers (Fig. S2).

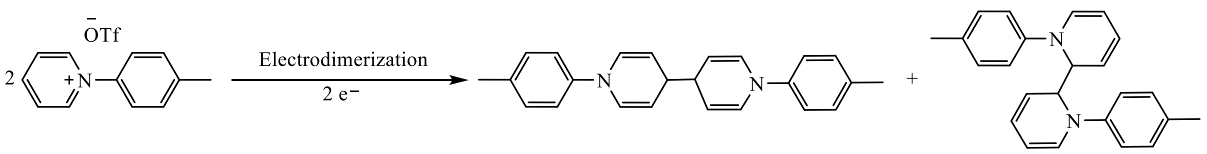

Supplementary Fig. S2. Molecular structures of dimers derived from electrochemical reduction of 1-(4-tolyl)pyridinium triflate salt.

The percentage of para,para dimer in the mixture could be quantified to constitute 71% from the specific multiplicity of the resonances between 6 and 6.5 ppm (doublet versus doublet of triplets) according to equation (S3).

$$\text{Percentage}_{\text{para,para}} = \frac{\text{integral (doublet/2)}}{\text{integral (double of triple) + integral (doublet/2)}} 100\% \qquad \text{(S3)}$$

SEM images of pristine Cu and Cu-n (n = 1–5) prior to CO_2RR

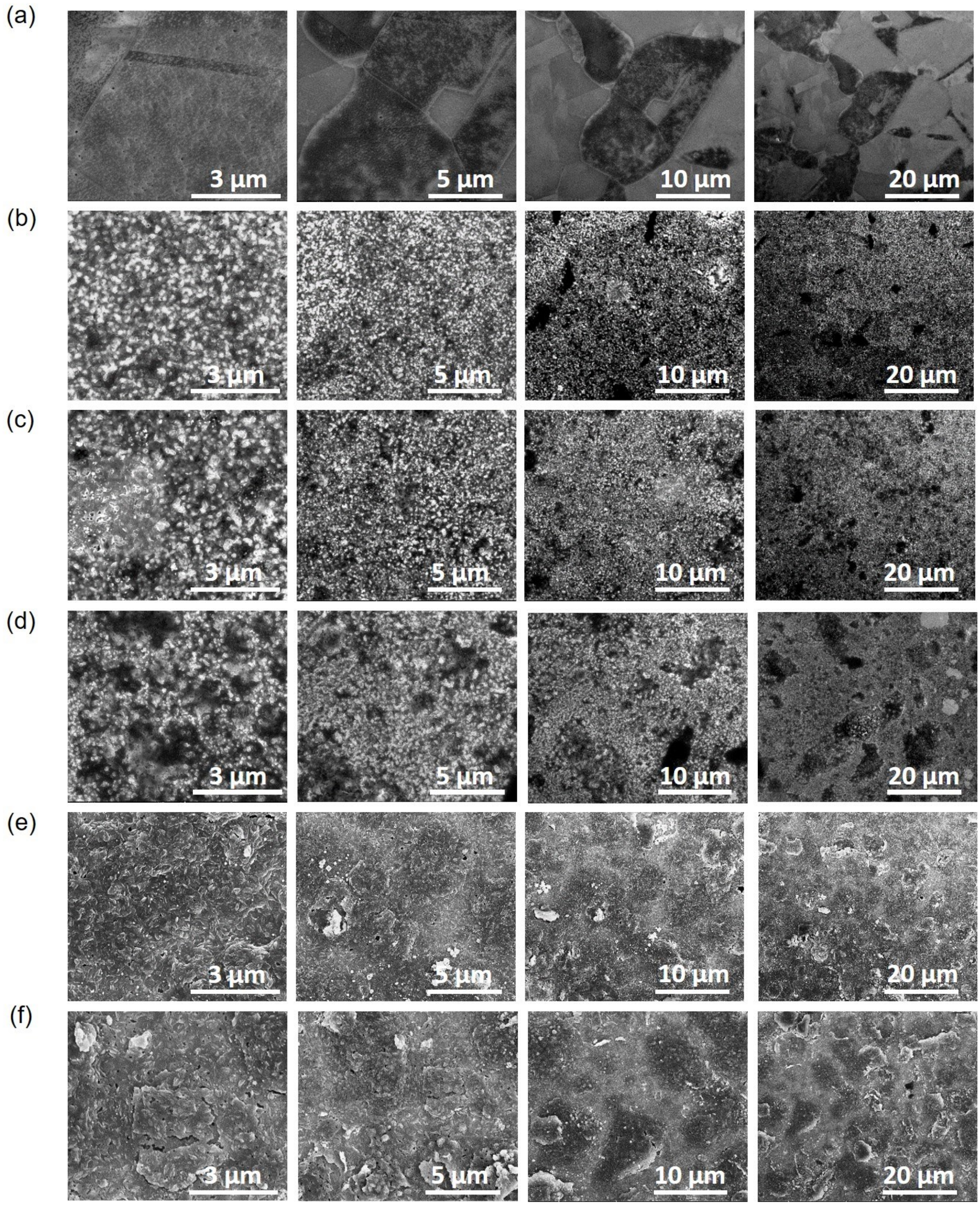

Supplementary Fig. S3. SEM images of (a) pristine Cu, (b) Cu-1, (c) Cu-2, (d) Cu-3, (e) Cu-4, and (f) Cu-5 electrodes at different magnifications prior to CO_2RR.

SEM images of electrografted Cu/T-bipyridine film electrode

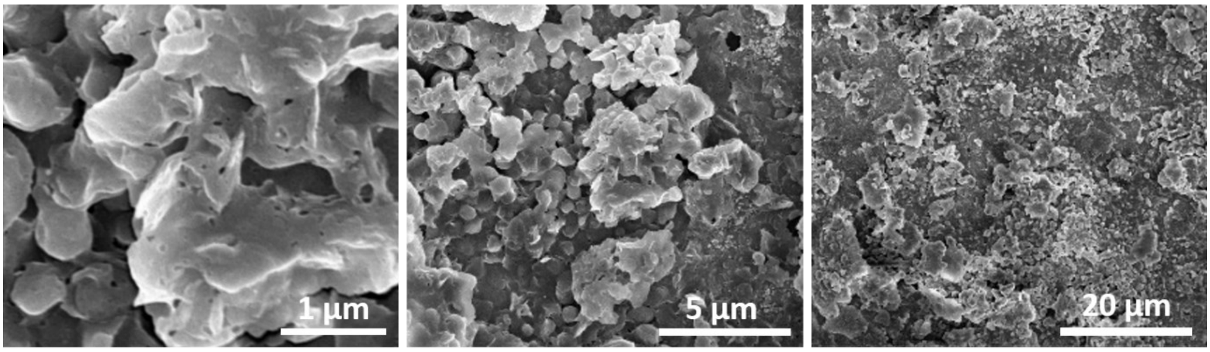

Supplementary Fig. S4. SEM images of Cu/T-bipyridine film electrode prepared from 10 mM 1-(4-tolyl)pyridinium triflate salt at an applied potential of –2.0 V versus RHE for 3600 s in CO_2-saturated 0.1 M $KHCO_3$ (pH = 6.8).

Optical micrograph of electrografted Cu/T-bipyridine film electrode

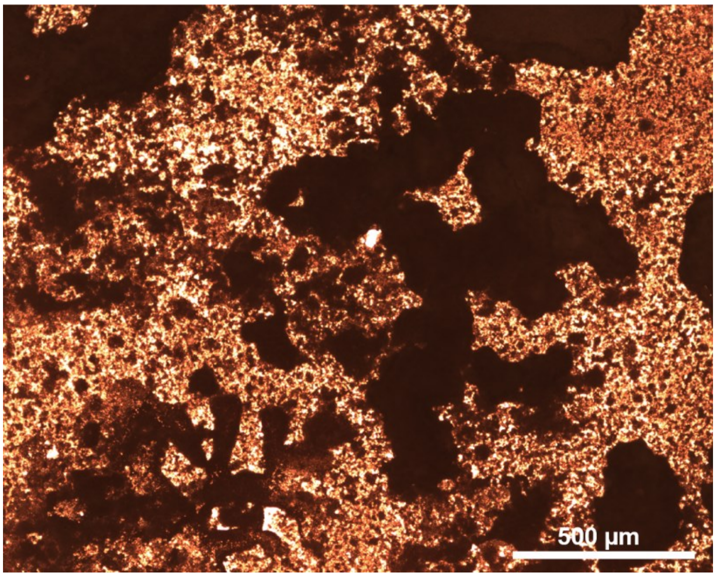

Supplementary Fig. S5. Optical micrograph of Cu/T-bipyridine film electrode prepared from 10 mM 1-(4-tolyl)pyridinium triflate salt at an applied potential of –2.0 V versus RHE for 3600 s in CO_2-saturated 0.1 M $KHCO_3$ (pH = 6.8).

ECSA measurements

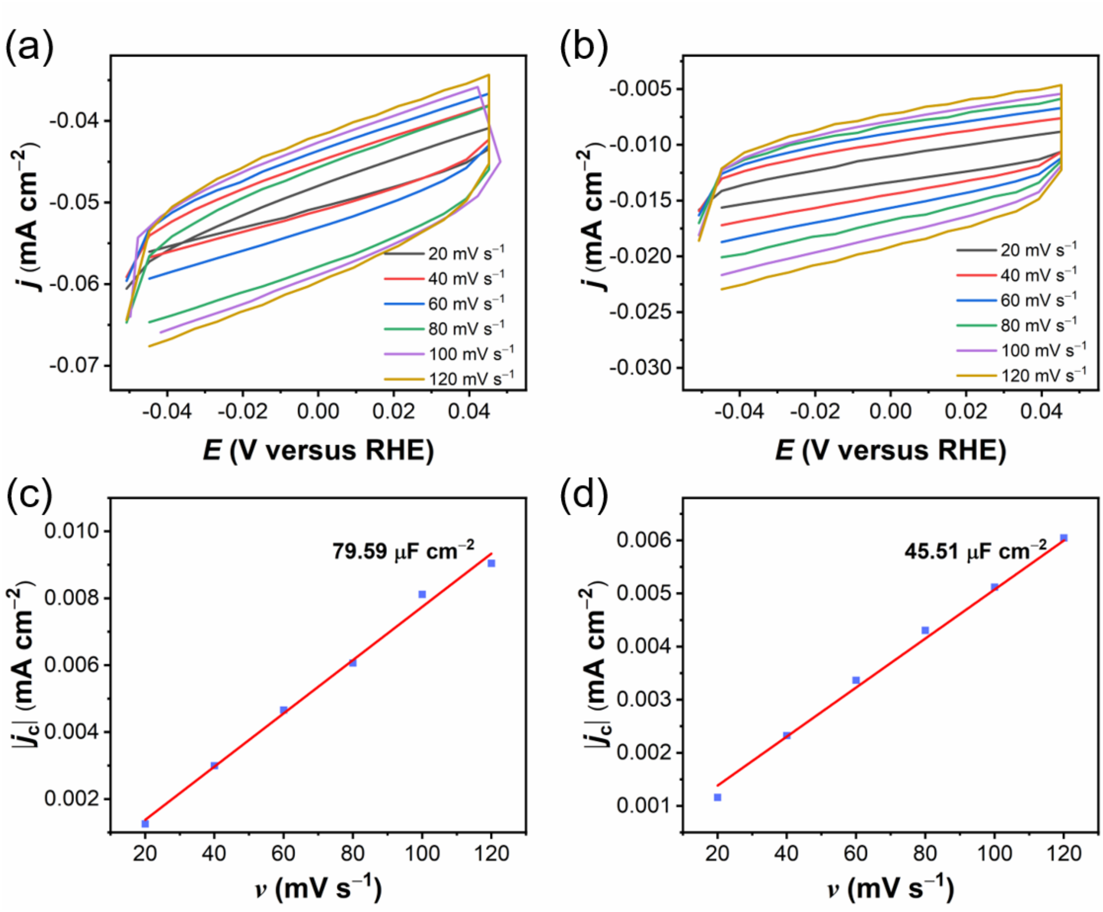

Supplementary Fig. S6. Cyclic voltammograms recorded on (a) pristine Cu and (b) Cu-5 electrodes in a non-faradaic range between –0.05 and 0.05 V versus RHE using various v's from 20–120 mV s^{-1} in CO_2-saturated 0.1 M $KHCO_3$ (pH = 6.8) prior to bulk electrolysis. Values of absolute electrochemical double layer capacitive current density, $|j_c|$, plotted against v for (c) pristine Cu and (d) Cu-5 electrodes, noting that $|j_c|$ was obtained by averaging the cathodic and anodic current densities at 0 V versus RHE from cyclic voltammograms in (a,b).

Pb underpotential deposition (Pb UPD)

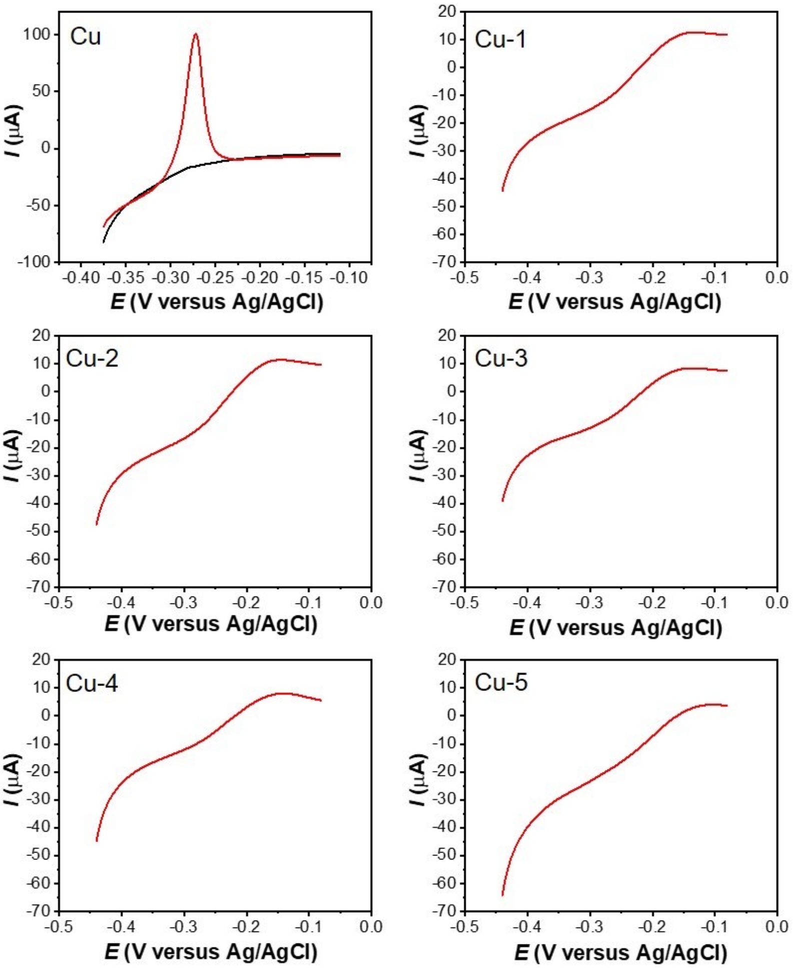

Supplementary Fig. S7. Linear sweep voltammetric curves of pristine Cu and Cu-n (n = 1–5) using v =10 mV s^{-1} after 10 min Pb deposition at –0.44 V vs Ag/AgCl in 0.1 M $HClO_4$ + 2 mM $Pb(ClO_4)_2$ aqueous solution.

We employed the Pb underpotential deposition method in an attempt to determine the ECSAs for all electrodes,[6,7] but it presented significant challenges for the Cu-n electrodes due to the presence of the organic films shifting the stripping curves in a positive potential direction to make it collide with the oxidation of bulk Cu itself (Fig. S7). This makes the integration of the areas underneath the curves uncertain, also because of problems of establishing appropriate baselines, thus preventing a quantitative assessment. To circumvent this problem, we tried to employ v < 10 mV s^{-1} during stripping, but also this had to be abandoned because of baseline problems. With these precautions taken, nevertheless, the size of the stripping curves seems to decrease with the size of the organic film as judged from Fig. S7, thus corroborating the values of ECSAs determined from the capacitive current measurements. Still, the main conclusion is that the Pb underpotential deposition method is inadequate for surface area determinations once the Cu electrode is covered with a thick organic film.

Partial current density ratio of C_2H_4/CH_4

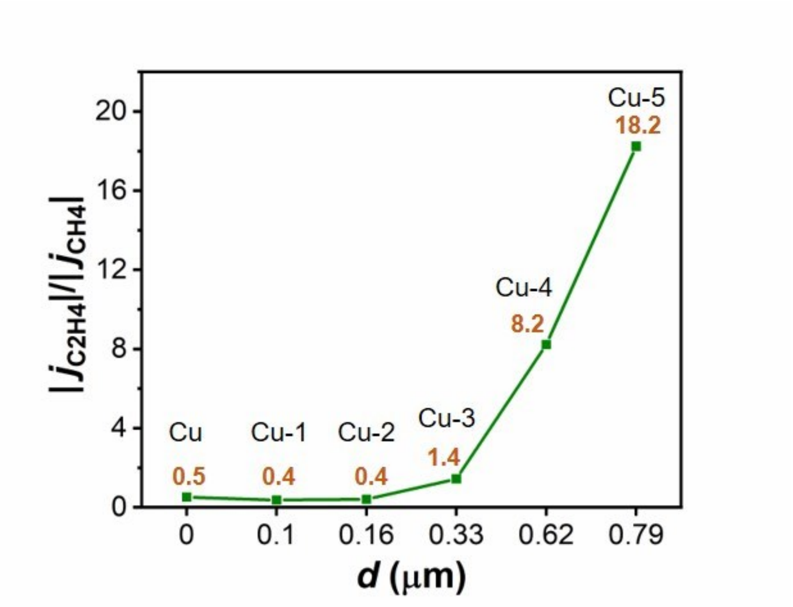

Supplementary Fig. S8. Partial current density ratio of C_2H_4 and CH_4, $|j_{C2H4}|/|j_{CH4}|$, as function of film thickness (d).

AFM images of pristine Cu and Cu-5 after removal of electrografted T-bipyridine film

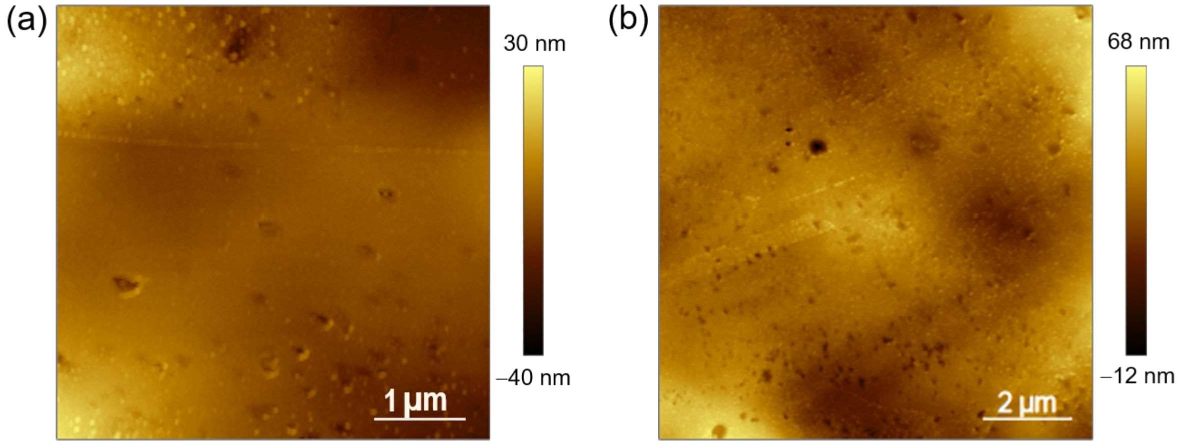

Supplementary Fig. S9. AFM images of (a) pristine Cu and (b) Cu-5 electrodes after removal of electrografted T-bipyridine film prepared from 10 mM 1-(4-tolyl)pyridinium triflate salt at an applied potential of −1.85 V versus RHE for 3600 s in CO_2-saturated 0.1 M $KHCO_3$ (pH = 6.8).

SEM images of pristine Cu and Cu-5 after removal of electrografted T-bipyridine film

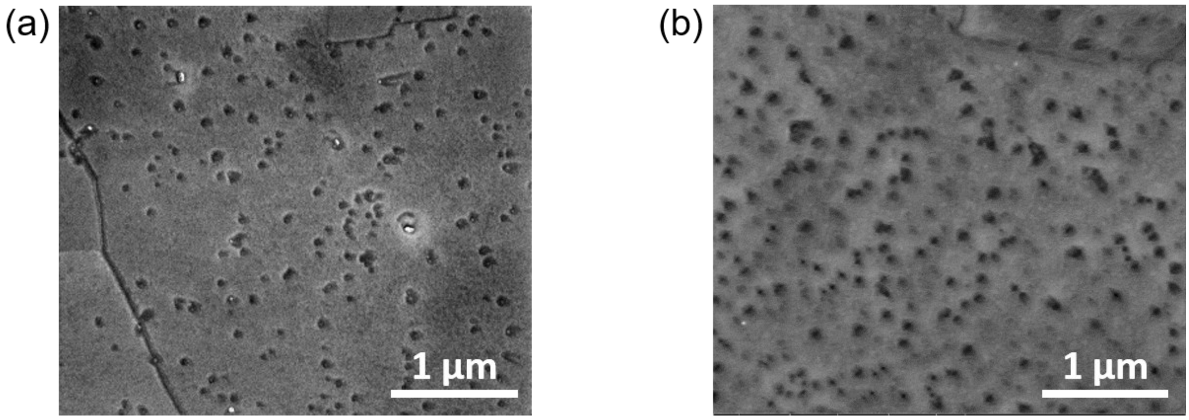

Supplementary Fig. S10. SEM images of (a) pristine Cu and (b) Cu-5 electrodes after removal of T-bipyridine film prepared from 10 mM 1-(4-tolyl)pyridinium triflate salt at an applied potential of −1.25 V versus RHE for 3600 s in CO_2-saturated 0.1 M $KHCO_3$ (pH = 6.8).

XPS spectra of pristine Cu and Cu-5 prior to CO_2RR

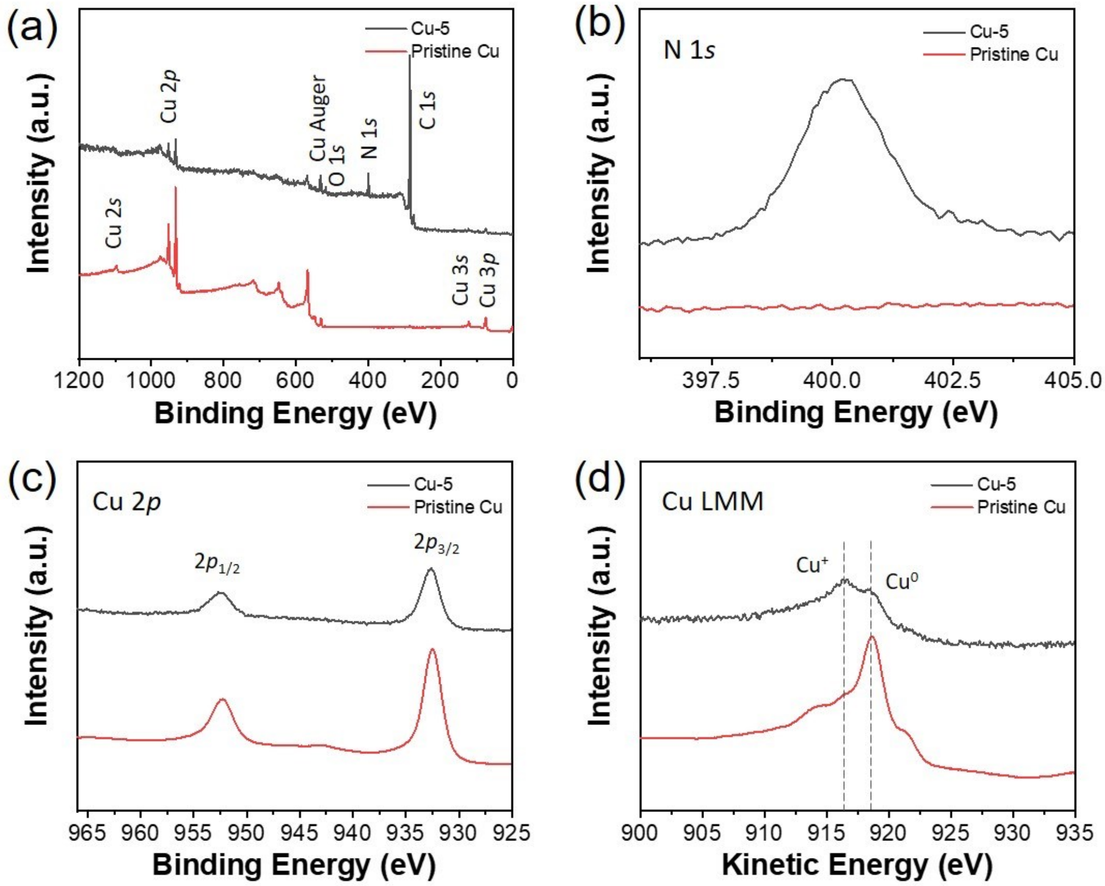

Supplementary Fig. S11. XPS spectra of (a) survey, (b) N 1*s*, (c) Cu 2*p* and (d) Cu LMM Auger for pristine Cu (red) and Cu-5 (gray) electrodes prior to CO_2RR.

SEM images of Cu-5$_{thin}$ prior to CO_2RR

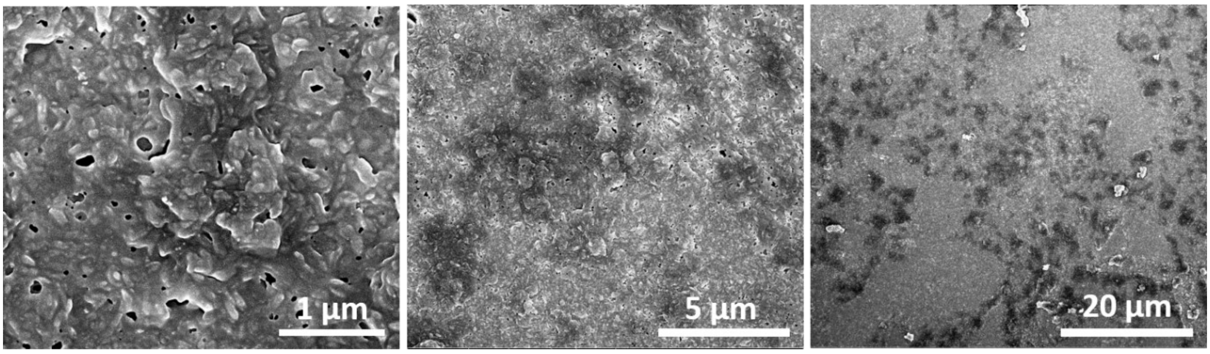

Supplementary Fig. S12. SEM images of Cu-5$_{thin}$ electrode with different magnifications prior to CO_2RR.

AFM image of Cu-5$_{thin}$ prior to CO_2RR

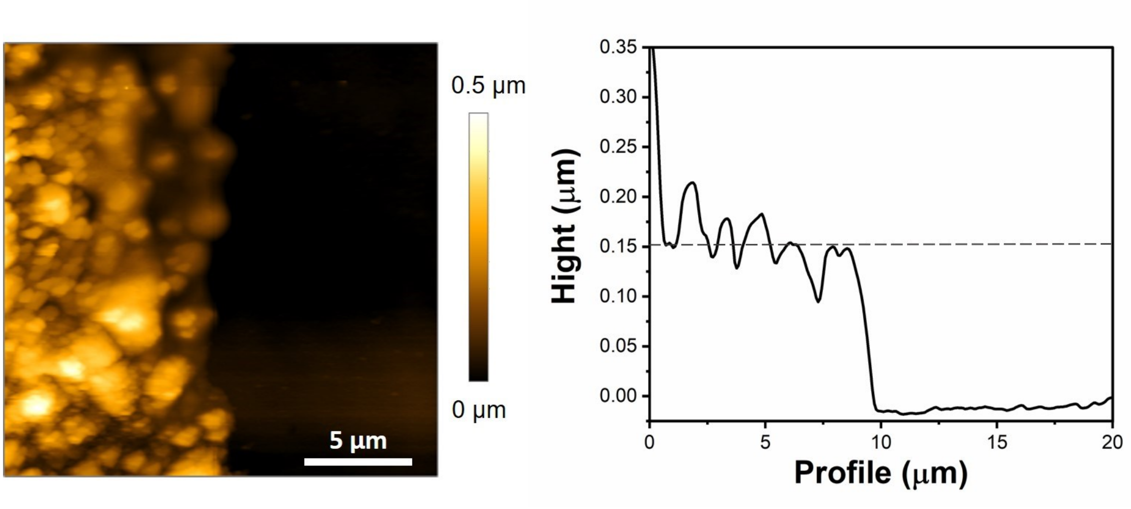

Supplementary Fig. S13. AFM image of Cu-5$_{thin}$ electrode prior to CO_2RR.

SEM images of Cu-5$_{block}$ prior to CO_2RR

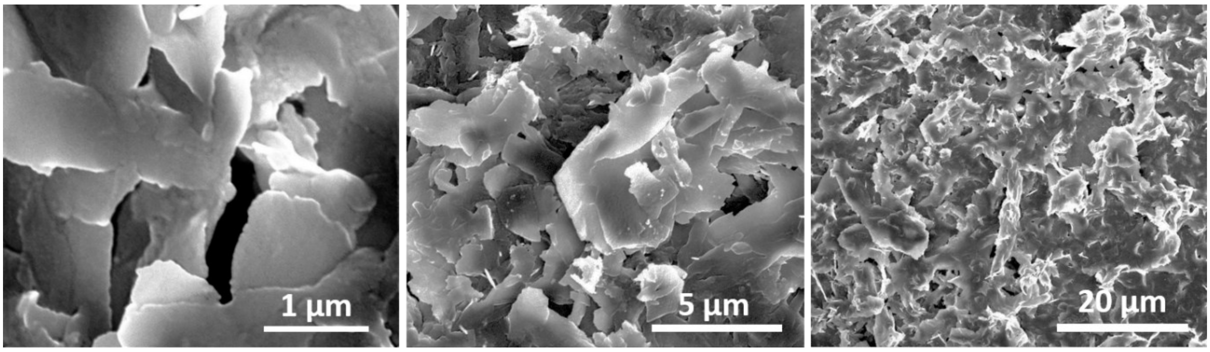

Supplementary Fig. S14. SEM images of Cu-5$_{block}$ electrode with different magnifications prior to CO_2RR.

AFM image of Cu-5$_{block}$ prior to CO_2RR

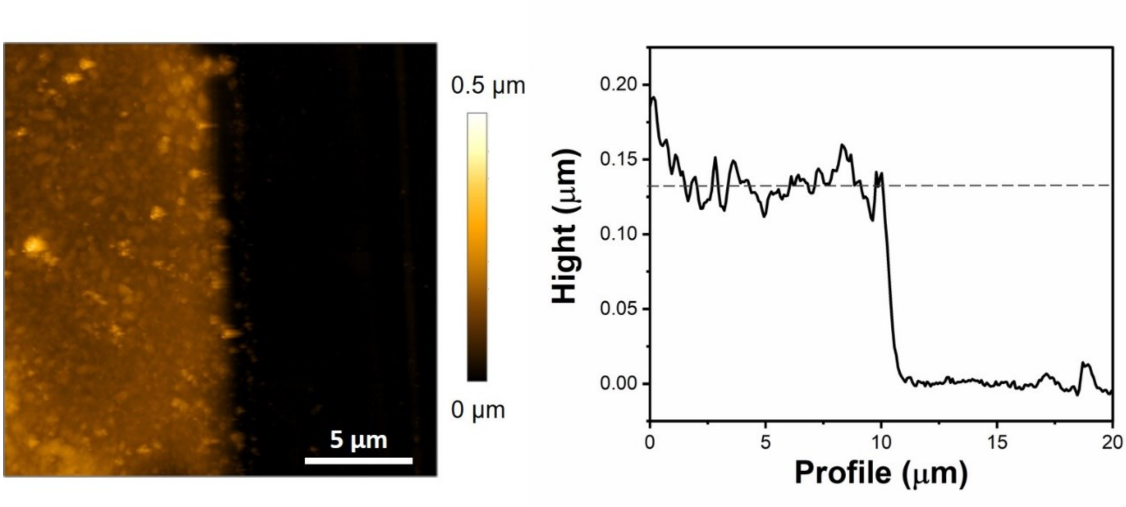

Supplementary Fig. S15. AFM image of Cu-5$_{block}$ electrode prior to CO_2RR.

Cyclic voltammogram of methyl viologen on pristine Cu

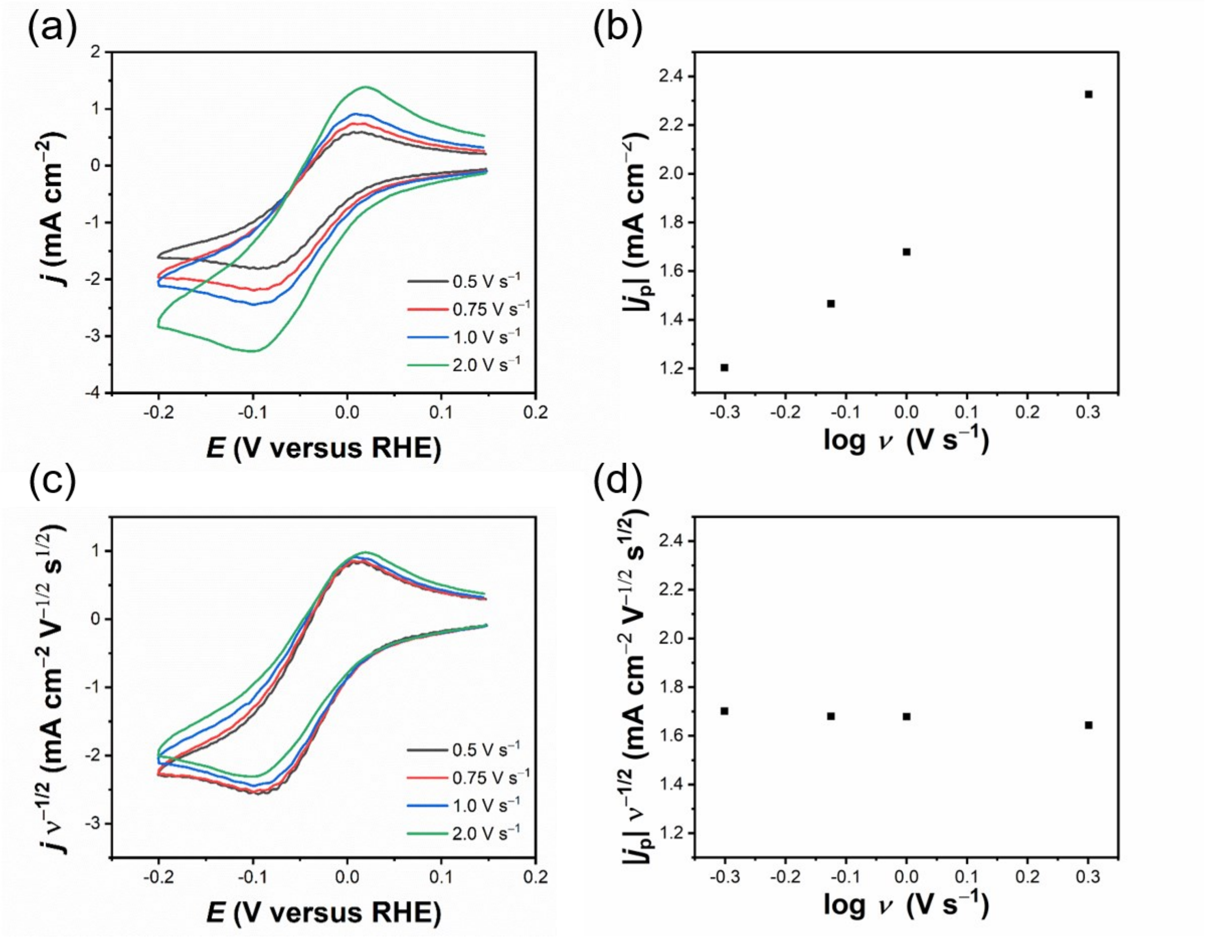

Supplementary Fig. S16. (a) Cyclic voltammograms of 2 mM methyl viologen recorded on a pristine Cu electrode (area = 0.0314 cm^2) using various v´s from 0.5–2.0 V s^{-1} in Ar-saturated 0.1 M $KHCO_3$ (pH = 8.4). (b) Absolute peak current density, $|j_p|$, plotted as function of log v. (c) Cyclic voltammograms and (d) absolute peak current density normalized by $v^{-1/2}$.

Cyclic voltammogram of methyl viologen on pristine Cu and Cu-5

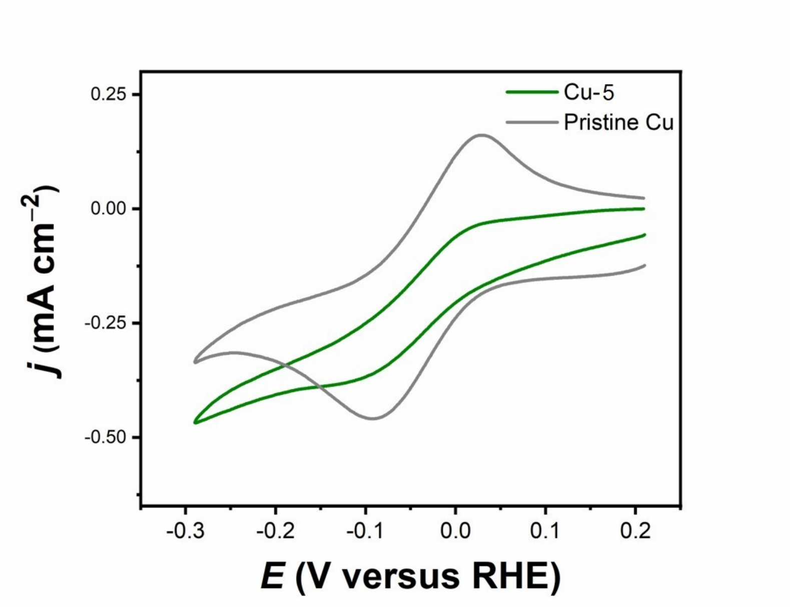

Supplementary Fig. S17. Cyclic voltammograms recorded on 2 mM methyl viologen on pristine Cu (gray) and Cu-5 (green) electrodes (area = 0.0314 cm^2) using v = 50 mV s^{-1} in Ar-saturated 0.1 M $KHCO_3$ (pH = 8.4).

Water contact angle for pristine Cu and Cu-n

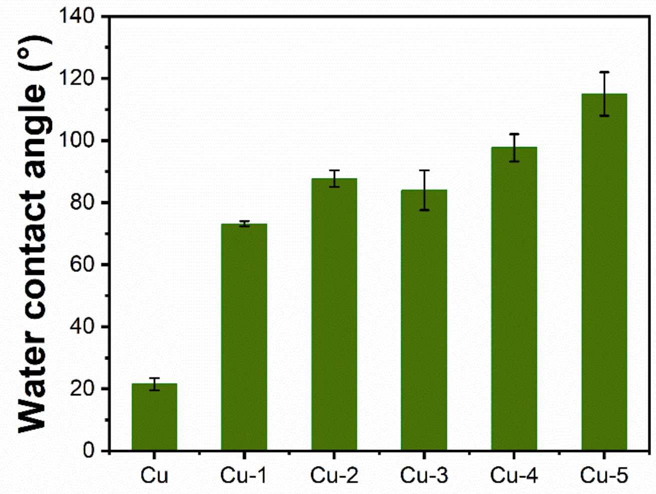

Supplementary Fig. S18. Water contact angle measurements of pristine Cu and Cu-n (n = 1–5) electrodes. Error bars correspond to the standard deviation of at least three independent measurements.

Operando Raman spectra of Cu and Cu-5 recorded at OCP

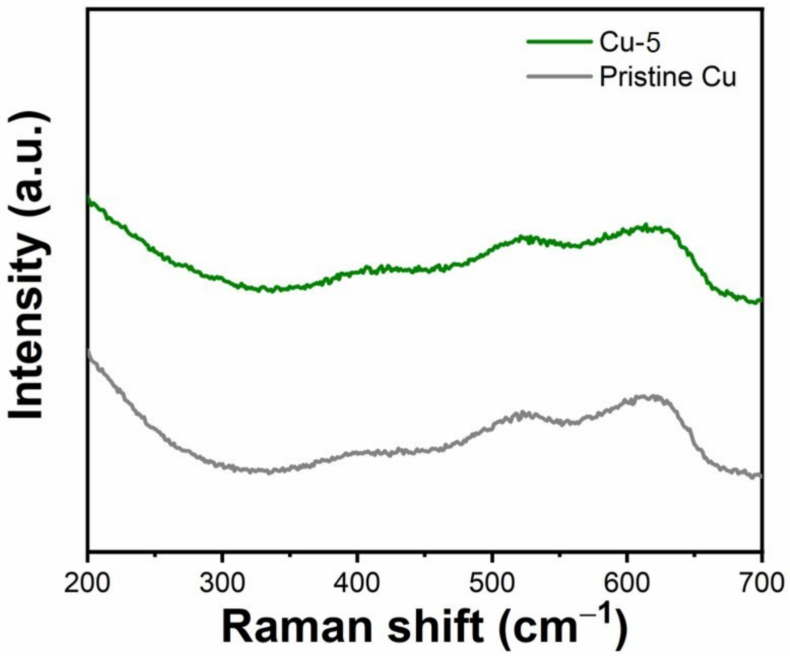

Supplementary Fig. S19. *Operando* Raman spectra recorded in the range of 200–700 cm^{-1} on pristine Cu (gray) and Cu-5 (green) electrodes at open circuit potential (OCP) in CO_2-saturated 0.1 M $KHCO_3$ (pH = 6.8).

Operando Raman spectra of Cu and Cu-5 recorded at –0.2 V versus RHE

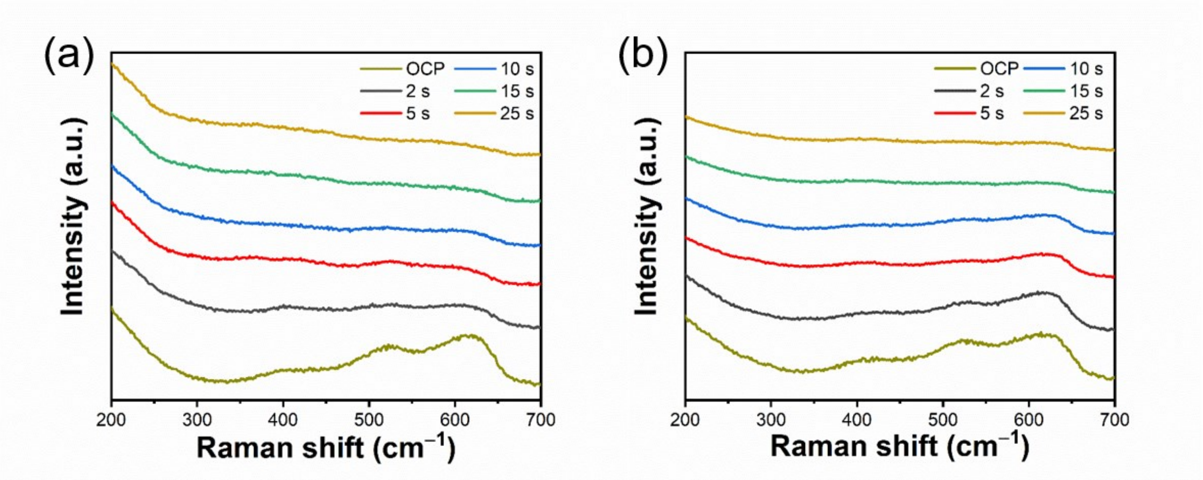

Supplementary Fig. S20. *Operando* Raman spectra recorded in the range of 200–700 cm^{-1} on (a) pristine Cu and (b) Cu-5 electrodes after different time periods of electroreduction at –0.2 V versus RHE in CO_2-saturated 0.1 M $KHCO_3$ (pH = 6.8).

Fig. S20 shows Raman spectra taken while applying a potential of –0.2 V versus RHE for different time periods. As seen, the Cu_2O related peaks are not present after 25 s of electroreduction, in line with Cu_2O being reduced into metallic Cu at the surface.

Microkinetic model for greater P_C and P_H

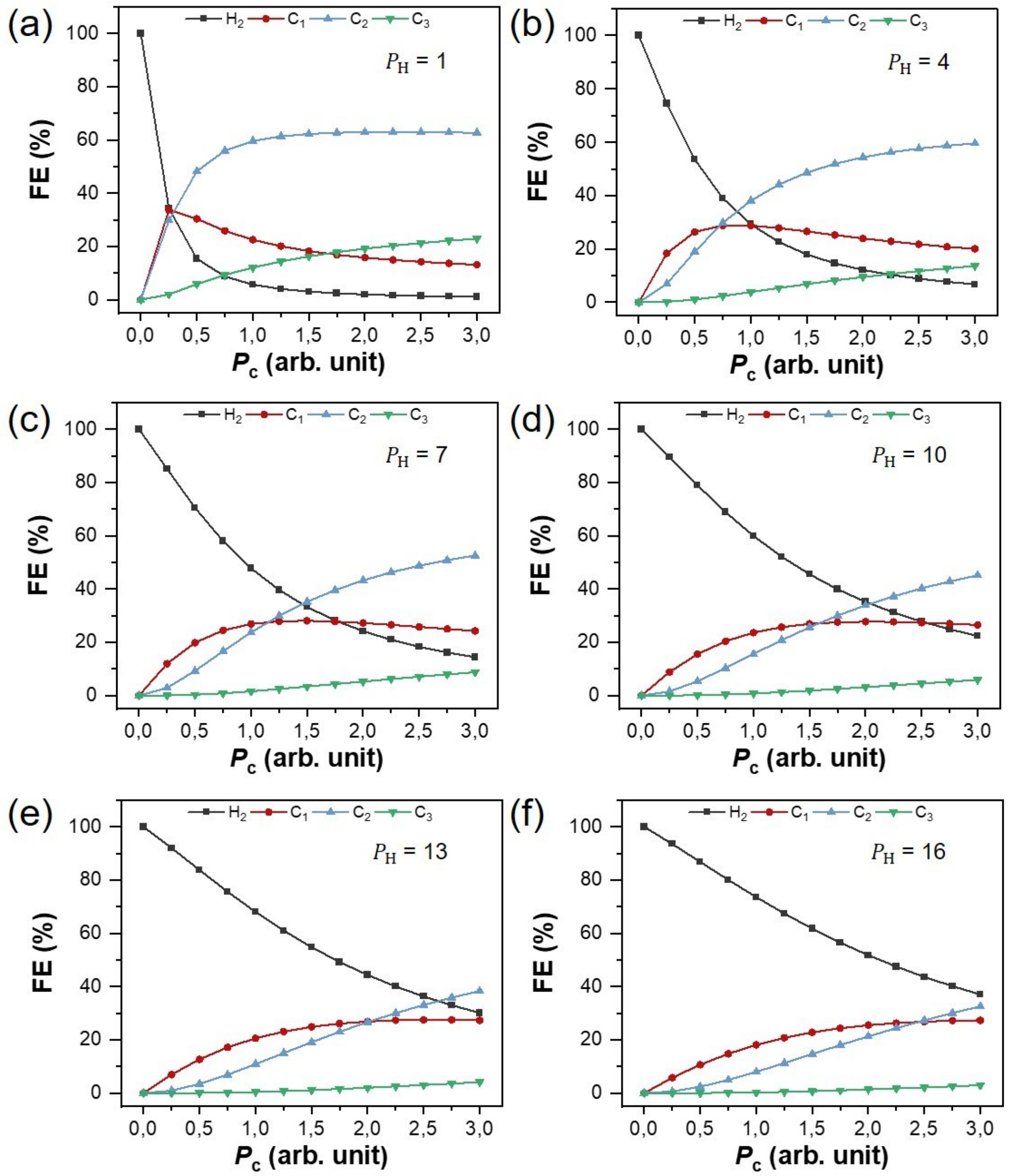

Supplementary Fig. S21. FEs of H_2 (black), C_1 (red), C_2 (blue), and C_3 (green) calculated from the model as function of carbon partial pressure P_C (arb. unit) at the surface under varying hydrogen partial pressure, P_H (arb. unit), with P_H = (a) 1, (b) 4, (c) 7, (d) 10, (e) 13, and (f) 16.

^{1}H NMR spectra of synthesized molecules

(a)

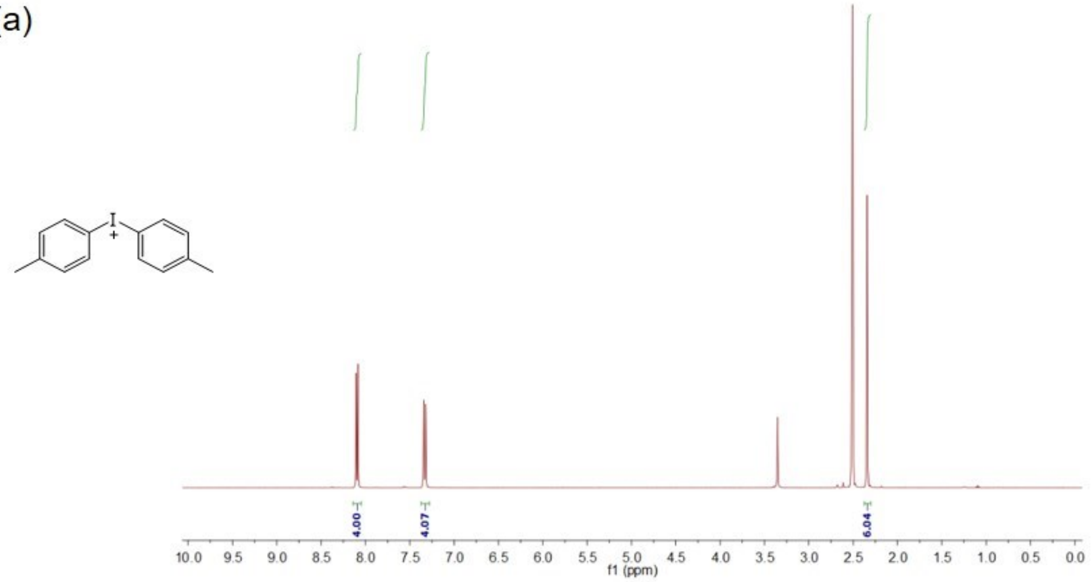

(b)

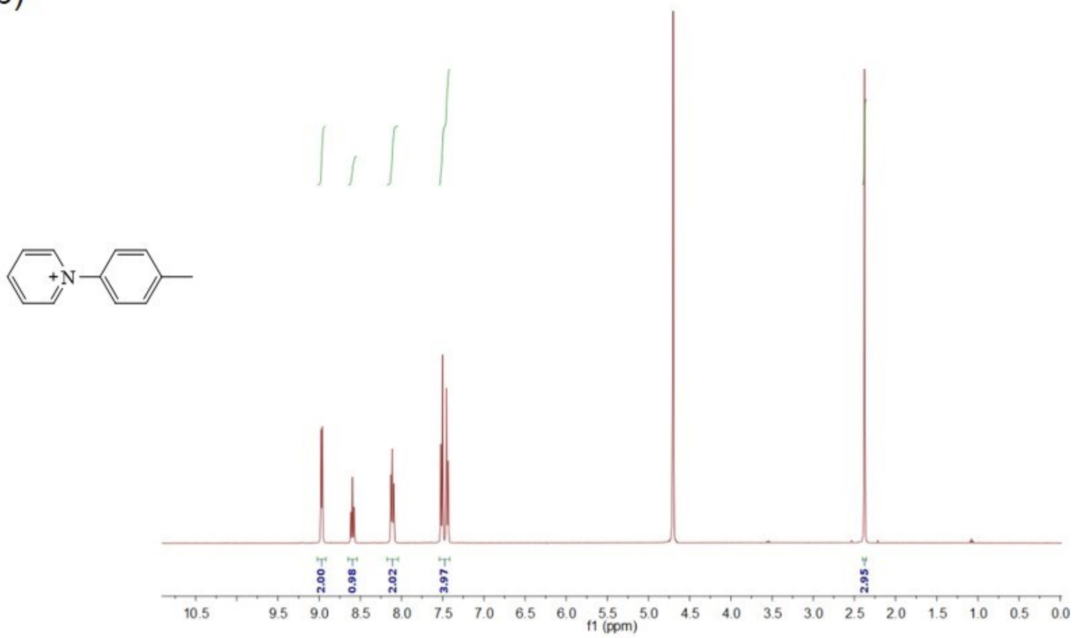

Supplementary Fig. S22. ^{1}H NMR spectra of (a) bis(4-tolyl)iodonium triflate salt and (b) 1-(4-tolyl)pyridinium triflate salt.

^{1}H NMR spectrum of dimer molecule obtained after electrodimerization of 1-(4-tolyl)pyridinium triflate salt

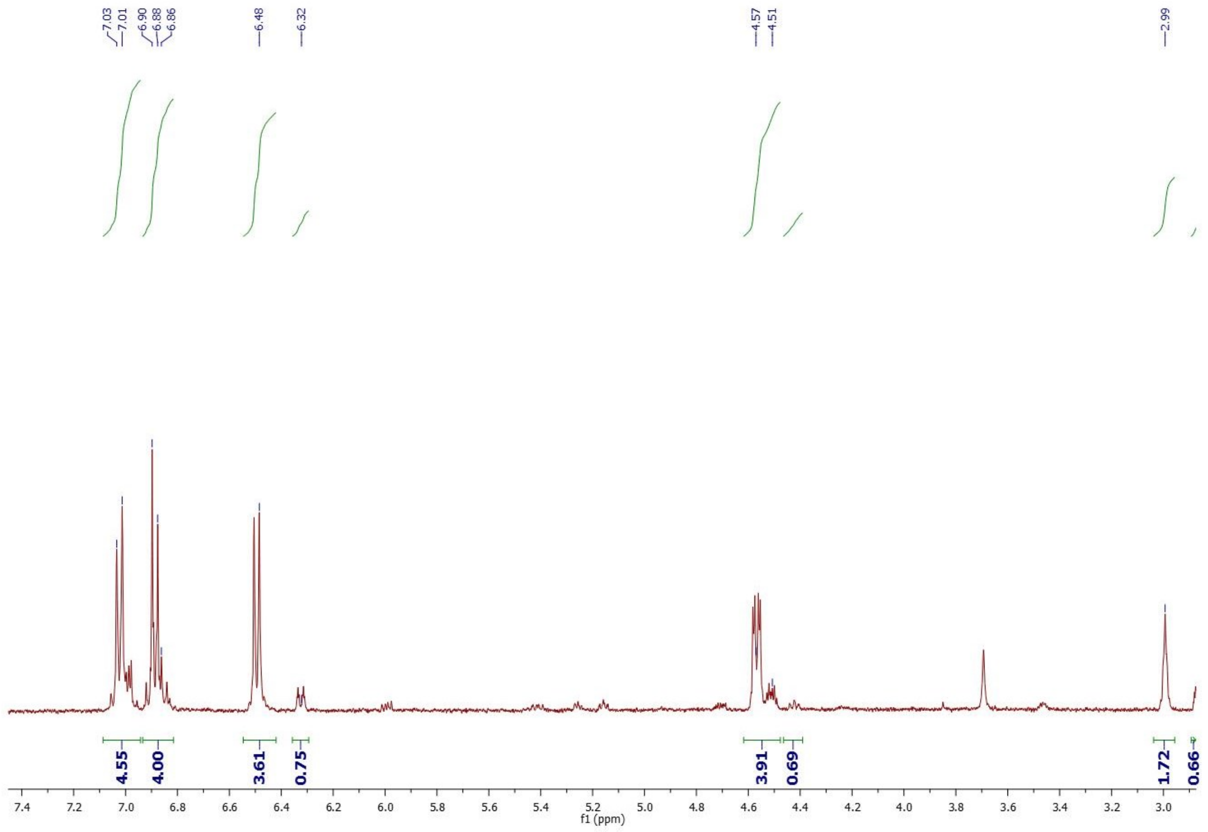

Supplementary Fig. S23. ^{1}H NMR spectrum of the organic film pertaining to electrodimerization of 1-(4-tolyl)pyridinium triflate salt, extracted from the copper electrode after electrochemical deposition.

Cyclic voltammogram of 1-(4-tolyl)pyridinium triflate salt

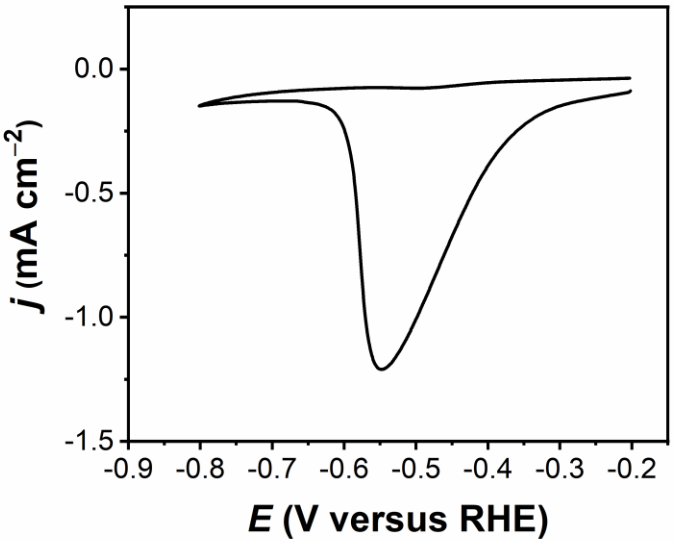

Supplementary Fig. S24. Cyclic voltammogram of 10 mM 1-(4-tolyl)pyridinium triflate salt in CO_2-saturated 0.1 M $KHCO_3$ (pH = 6.8).

Operando Raman electrochemical cell

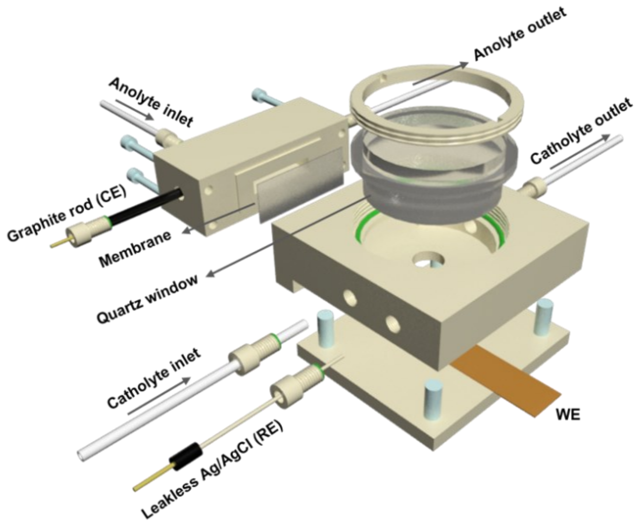

Supplementary Fig. S25. Diagrams showing buildup of *operando* Raman electrochemical cell.

Table of deposition conditions of Cu-n

Table S1. Deposition Conditions of T-bipyridine Modified Cu Electrodes.[a]

	E (V versus RHE)	Deposition time (s)
Cu-1	−0.55	300
Cu-2	−0.70	900
Cu-3	−0.10	900
Cu-4	−1.20	3600
Cu-5	−1.25	3600

[a]Electrodes were prepared from 10 mM 1-(4-tolyl)pyridinium triflate salt in CO_2-saturated 0.1 M $KHCO_3$ (pH = 6.8).

Table of film porosity of Cu-n

Table S2. Film Porosity, P, of Cu-n (n = 1–5) Electrodes. [a]

	d (µm)	d_{recon} (µm)	V_{pore} (cm^3)	V_{total} (cm^3)	P (%)
Cu-1	0.10 ± 0.01	0.05 ± 0.01	6.71×10^{-6}	1.22×10^{-5}	55
Cu-2	0.16 ± 0.02	0.08 ± 0.03	9.76×10^{-6}	1.95×10^{-5}	50
Cu-3	0.33 ± 0.05	0.11 ± 0.01	2.68×10^{-5}	4.03×10^{-5}	67
Cu-4	0.62 ± 0.06	0.13 ± 0.01	6.04×10^{-5}	7.56×10^{-5}	80
Cu-5	0.79 ± 0.08	0.13 ± 0.02	8.05×10^{-5}	9.82×10^{-5}	83

[a] P is calculated according to equation (1), where it for the sake of completeness may be noted that $A = 1.22$ cm^2.

Table of CO_2RR performance on pristine Cu and Cu-n

Table S3. CO_2RR Performance Obtained on Pristine Cu and Cu-n (n = 1–5).[a]

		$\|j_{geo}\|$[b] (mA cm^{-2})	C_{DL} (μF cm^{-2})	RF[c]	$\|j_{ECSA}\|$[d] (mA cm^{-2})	FE_{H2} (%)	FE_{CH4} (%)	FE_{CO} (%)	FE_{C2H4} (%)	FE_{HCOO-} (%)	$FE_{CH3COO-}$ (%)	FE_{C2H5OH} (%)	$FE_{n\text{-}C3H7OH}$ (%)	FE_{total} (%)
Cu	Entry-1	2.7	79.6	2.7	1.0	56.6	13.0	12.9	7.2	7.8	0.6	1.4	2.3	101.8
	Entry-2	2.6	70.1	2.4	1.1	40.7	16.9	11.1	9.1	10.5	0.9	2.1	2.3	93.6
	Entry-3	2.6	79.1	2.7	1.0	49.4	13.6	2.8	6.3	4.9	0.5	1.3	1.9	80.7
	Entry-4	2.1	64.7	2.2	1.1	64.4	5.8	10.4	2.5	6.8	0.6	1.0	ND[e]	91.5
	Entry-5	2.4	72.8	2.5	0.9	52.9	9.8	9.7	5.6	10.3	0.7	1.2	1.0	91.2
	Average	2.5 ± 0.2	73.3 ± 6.3	2.5 ± 0.2	1.0 ± 0.1	52.8 ± 8.8	11.8 ± 4.2	9.4 ± 3.9	6.1 ± 2.4	8.1 ± 2.4	0.7 ± 0.2	1.4 ± 0.4	1.5 ± 1.0	91.8 ± 7.5
Cu-1	Entry-1	2.4	68.3	2.4	1.0	55.3	12.7	6.9	5.2	6.6	0.4	1.7	1.1	89.9
	Entry-2	2.4	65.1	2.3	1.1	53.5	15.2	7.3	5.0	5.2	0.4	2.6	1.6	90.8
	Average	2.4 ± 0.0	66.7± 2.3	2.3 ± 0.1	1.1 ± 0.1	54.4 ± 1.3	14.0 ± 1.8	7.1 ± 0.3	5.1 ± 0.1	5.9 ± 1.0	0.4 ± 0.0	2.2 ± 0.6	1.4 ± 0.4	90.4 ± 0.6
Cu-2	Entry-1	2.4	75.8	2.6	0.9	46.4	21.7	6.2	9.2	4.2	0.3	3.0	1.4	92.4
	Entry-2	2.2	47.0	1.6	1.4	45.8	22.9	7.5	8.8	6.0	0.4	1.6	1.2	94.2
	Average	2.3 ± 0.1	61.4 ± 20.4	2.1 ± 0.7	1.2 ± 0.4	46.1 ± 0.4	22.3 ± 0.8	6.9 ± 0.9	9.0 ± 0.3	5.1 ± 1.3	0.4 ± 0.1	2.3 ± 1.0	1.3 ± 0.1	93.4 ± 1.2
Cu-3	Entry-1	2.4	52.0	1.8	1.3	53.4	12.3	4.6	10.0	3.2	0.4	4.9	1.9	90.7
	Entry-2	2.5	63.0	2.2	1.1	53.1	3.9	2.0	20.4	4.0	0.4	4.8	3.9	92.5
	Entry-3	2.3	64.3	2.2	1.0	38.1	17.8	6.7	18.3	4.4	0.6	3.7	2.7	92.2
	Average	2.4 ± 0.1	59.8 ± 6.8	2.1 ± 0.2	1.1 ± 0.1	48.2 ± 8.7	11.3 ± 7.0	4.4 ± 2.4	16.2 ± 5.5	3.9 ± 0.6	0.5 ± 0.1	4.5 ± 0.7	2.8 ± 1.0	91.8 ± 1.0
Cu-4	Entry-1	2.4	52.6	1.8	1.3	40.5	7.1	4.6	26.6	3.5	0.5	5.6	3.6	92.0
	Entry-2	1.9	40.7	1.4	1.4	29.8	2.0	10.9	27.5	5.1	0.6	7.6	5.2	88.7
	Entry-3	2.1	50.9	1.8	1.2	39.5	1.5	2.3	32.8	3.8	0.5	8.5	4.3	93.2
	Average	2.1 ± 0.3	48.1 ± 6.4	1.7 ± 0.2	1.3 ± 0.1	36.6 ± 5.9	3.5 ± 3.1	5.9 ± 4.5	29.0 ± 3.4	4.1 ± 0.9	0.5 ± 0.1	7.2 ± 1.5	4.4 ± 0.8	91.3 ± 2.3
Cu-5	Entry-1	1.9	45.5	1.6	1.2	23.6	2.7	ND[e]	42.3	5.1	0.7	10.7	5.6	90.7
	Entry-2	1.8	50.2	1.7	1.1	25.7	2.4	ND[e]	49.9	2.5	0.6	10.9	3.0	95.0
	Average	1.9 ± 0.1	47.9 ± 3.3	1.7 ± 0.1	1.1 ± 0.1	24.7 ± 1.5	2.6 ± 0.2	ND[e]	46.1 ± 5.4	3.8 ± 1.8	0.7 ± 0.1	10.8 ± 0.2	4.3 ± 1.8	92.9 ± 3.0

[a]Applied potential = –0.96 V versus RHE in CO_2-saturated 0.1 M $KHCO_3$ (pH = 6.8). [b]Absolute current density normalized with respect to geometric area (= 1.22 cm^2). [c]Roughness factor. [d]Absolute current density normalized with respect to ECSA (i.e. $|j_{ECSA}| = |j_{geo}|/RF$). [e] Not detected.

Table of HER performance on pristine Cu and Cu-n

Table S4. HER Performance Obtained on Pristine Cu and Cu-n (n = 1–5).[a]

		$\|j_{geo}\|$[b] (mA cm^{-2})	C_{DL} (µF cm^{-2})	RF[c]	$\|j_{ECSA}\|$[d] (mA cm^{-2})
Cu	Entry-1	2.7	72.5	2.5	1.1
	Entry-2	2.7	78.3	2.7	1.0
	Average	2.7 ± 0.0	75.4 ± 4.1	2.6 ± 0.1	1.1 ± 0.1
Cu-1	Entry-1	2.6	81.2	2.8	0.9
	Entry-2	2.9	72.5	2.5	1.2
	Average	2.8 ± 0.2	76.9 ± 6.2	2.7 ± 0.2	1.1 ± 0.2
Cu-2	Entry-1	2.5	66.7	2.3	1.1
	Entry-2	2.5	60.9	2.1	1.2
	Average	2.5 ± 0.0	65.3 ± 2.1	2.3 ± 0.1	1.2 ± 0.1
Cu-3	Entry-1	2.2	60.9	2.1	1.1
	Entry-2	2.3	60.9	2.0	1.2
	Average	2.3 ± 0.1	60.9 ± 0.0	2.1 ± 0.0	1.2 ± 0.1
Cu-4	Entry-1	2.1	58.0	2.0	1.1
	Entry-2	1.9	52.2	1.8	1.1
	Average	2.0 ± 0.1	55.1 ± 4.1	1.9 ± 0.1	1.1 ± 0.0
Cu-5	Entry-1	1.7	55.1	1.9	0.9
	Entry-2	2.1	52.6	1.8	1.2
	Average	1.9 ± 0.3	53.9 ± 1.8	1.9 ± 0.1	1.1 ± 0.2

[a]Applied potential = –0.96 V versus RHE in CO_2-saturated 0.1 M $KHCO_3$ (pH = 6.8). [b]Absolute current density normalized with respect to geometric area (= 1.22 cm^2). [c]Roughness factor. [d]Absolute current density normalized with respect to ECSA (i.e. $|j_{ECSA}| = |j_{geo}|/RF$).

Table of CO_2RR performance on Cu-5$_{thin}$

Table S5. CO_2RR Performance Obtained on Cu-5$_{thin}$.[a]

		$\|j_{geo}\|$[b] (mA cm^{-2})	C_{DL} (μF cm^{-2})	RF[c]	$\|j_{ECSA}\|$[d] (mA cm^{-2})	FE_{H2} (%)	FE_{CH4} (%)	FE_{CO} (%)	FE_{C2H4} (%)	FE_{HCOO-} (%)	$FE_{CH3COO-}$ (%)	FE_{C2H5OH} (%)	$FE_{n-C3H7OH}$ (%)	FE_{total} (%)
Cu-5$_{thin}$	Entry-1	2.4	66.2	2.3	1.0	36.2	5.3	4.7	25.2	6.2	*	4.3	3.4	85.3
	Entry-2	2.2	57.8	2.0	1.1	39.3	5.8	4.9	27.3	4.7	0.2	5.2	3.5	90.8
	Average	2.3 ± 0.1	62.0 ± 5.9	2.1 ± 0.2	1.1 ± 0.1	37.8 ± 2.2	5.6 ± 0.4	4.8 ± 0.1	26.3 ± 1.5	5.5 ± 1.1	0.1 ± 0.1	4.8 ± 0.6	3.5 ± 0.1	88.1 ± 4.0

[a]Applied potential = –0.96 V versus RHE in CO_2-saturated 0.1 M $KHCO_3$ (pH = 6.8). [b]Absolute current density normalized with respect to geometric area (= 1.22 cm^2). [c]Roughness factor. [d]Absolute current density normalized with respect to ECSA (i.e. $|j_{ECSA}| = |j_{geo}|/RF$).

Table of CO_2RR performance on Cu-5$_{block}$

Table S6. CO_2RR Performance Obtained on Cu-5$_{block}$.[a]

		$\lvert j_{geo}\rvert$[b] (mA cm^{-2})	C_{DL} (μF cm^{-2})	RF[c]	$\lvert j_{ECSA}\rvert$[d] (mA cm^{-2})	FE_{H2} (%)	FE_{CH4} (%)	FE_{CO} (%)	FE_{C2H4} (%)	FE_{HCOO-} (%)	$FE_{CH3COO-}$ (%)	FE_{C2H5OH} (%)	$FE_{n-C3H7OH}$ (%)	FE_{total} (%)
Cu-5$_{block}$	Entry-1	2.5	59.3	2.0	1.3	54.1	16.6	10.9	8.7	4.3	0.1	1.8	1.2	97.7
	Entry-2	2.4	64.1	2.2	1.1	49.9	10.0	7.1	6.9	8.4	0.6	1.4	1.5	85.8
	Average	2.5 ± 0.1	61.7 ± 3.4	2.1 ± 0.1	1.2 ± 0.1	52.0 ± 3.0	13.3 ± 4.7	9.0 ± 2.7	7.8 ± 1.3	6.4 ± 2.9	0.4 ± 0.4	1.6 ± 0.3	1.4 ± 0.2	91.8 ± 8.4

[a]Applied potential = –0.96 V versus RHE in CO_2-saturated 0.1 M $KHCO_3$ (pH = 6.8). [b]Absolute current density normalized with respect to geometric area (= 1.22 cm^2). [c]Roughness factor. [d]Absolute current density normalized with respect to ECSA (i.e. $\lvert j_{ECSA}\rvert = \lvert j_{geo}\rvert$/RF).

Supplementary References

1. Aristov, N. & Habekost, A. Electrochromism of Methylviologen (Paraquat). *World J. Chem. Educ.* **3**, 82–86 (2015).
2. Flory, P. J. Molecular Size Distribution in Linear Condensation Polymers [1]. *J. Am. Chem. Soc.* **58**, 1877–1885 (1936).
3. Kuhl, K. P., Cave, E. R., Abram, D. N. & Jaramillo, T. F. New insights into the electrochemical reduction of carbon dioxide on metallic copper surfaces. *Energy Environ. Sci.* **5**, 7050 (2012).
4. Li, F. *et al.* Molecular tuning of CO_2-to-ethylene conversion. *Nature* **577**, 509–513 (2020).
5. Thevenon, A., Rosas-Hernández, A., Fontani Herreros, A. M., Agapie, T. & Peters, J. C. Dramatic HER Suppression on Ag Electrodes via Molecular Films for Highly Selective CO_2 to CO Reduction. *ACS Catal.* **11**, 4530–4537 (2021).
6. Baturina, O. A. *et al.* CO_2 Electroreduction to Hydrocarbons on Carbon-Supported Cu Nanoparticles. *ACS Catal.* **4**, 3682–3695 (2014).
7. Plaza-Mayoral, E. *et al.* Preparation of high surface area Cu-Au bimetallic nanostructured materials by co-electrodeposition in a deep eutectic solvent. *Electrochimica Acta* **398**, 139309 (2021).
